# NCS-Mediated *Ipso*-Halogenation of Arylboronic Acids in Water Using Sodium Halides

**DOI:** 10.1021/acsomega.5c00755

**Published:** 2025-06-25

**Authors:** Alessandro Santarsiere, Pierantonio Galgano, Maria Funicello, Paolo Lupattelli, Lucia Chiummiento

**Affiliations:** † Department of Basic and Applied Sciences, University of Basilicata, Via dell’Ateneo lucano 10, Potenza 85100, Italy; ‡ Department of Chemistry, University “La Sapienza” of Roma, Piazzale A. Moro 5, Roma 00185, Italy

## Abstract

A mild, efficient, eco-friendly method for the regioselective halogenation of arylboronic acids in water has been reported. A transition-metal-free *ipso*-chlorination of arylboronic acids was performed in water, yielding aryl chlorides. *Ipso*-brominated and iodinated compounds were obtained under the same conditions using the corresponding halide salts. This eco-friendly method operates efficiently, even on activated or nonactivated systems, with good yields and selectivity.

## Graphical abstract

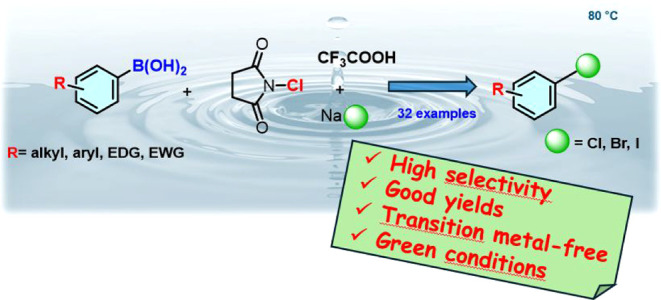

## Introduction

Aryl chlorides are present in over 200 FDA-approved drugs.^1^ They are essential components of many pharmacologically active compounds,^2^ capable of modifying pharmacokinetic profiles and, thus, pharmacological properties.^3^ Since they are also key synthetic intermediates,^4^ efficient and regioselective chlorination methodologies are of current synthetic interest. Traditionally, they can be prepared by electrophilic aromatic substitution^5^ or the Sandmeyer reaction.^6^ These methods often face challenges such as poor regio- and chemoselectivity, low yields, harsh reaction conditions, and long reaction times. Palladium-catalyzed regio- and chemoselective halogenations are successfully performed, as those reported by Rao^7^ on electron-deficient arenes with NCS or by Buchwald on aryl triflates,^8^ but the use of toxic and expensive palladium has limited their application. More recently, *ipso*-functionalization of boronic acids^9^ offered a site-specific substitution approach, effectively addressing the challenges of regio- and chemoselectivity that arise from traditional electrophilic halogenation. Nevertheless, various substituted arylboronic acids can be obtained through a directed C–H borylation method.^10^ Additionally, boronic acid can act as a protective group for aryl halides, especially for highly functionalized organic structures. While bromo- and iodo-arenes can be efficiently synthesized by direct halogenation with NBS and NIS,^9^ NCS proved to be inefficient in the absence of a copper-based catalyst (Scheme 1), likely due to the low reactivity of NCS.^11^ A preliminary study on *ipso*-chlorination with NCS was conducted under basic conditions in MeCN, yielding potentially interesting results despite being performed on only four substrates.^11^ Huffman reported that Cu­(II) can facilitate the bromination of arylboronates,^12^ and Hartwig later extended this method to chlorides.^13^ While copper-mediated chlorodeboronation of arylboronic acids or arylboronates using inexpensive NCS has proven highly efficient for producing aryl chlorides, the approach requires excess Cu­(II) halide salts (up to 3.5 equiv). Recently, a copper-catalyzed boron-chloride exchange was developed, but the removal of the copper salts from the reaction mixture remains challenging.^11^ As is well-known, homogeneous catalysis often leads to heavy metal contamination of the final product and limits its use in electronics and biomedicine. These issues pose significant environmental and economic concerns, particularly for large-scale industrial syntheses. To address these challenges, this work describes a transition-metal-free *ipso*-chlorination using NCS, with or without NaCl, in water, which operates efficiently even on activated or deactivated systems.

**Scheme 1:**
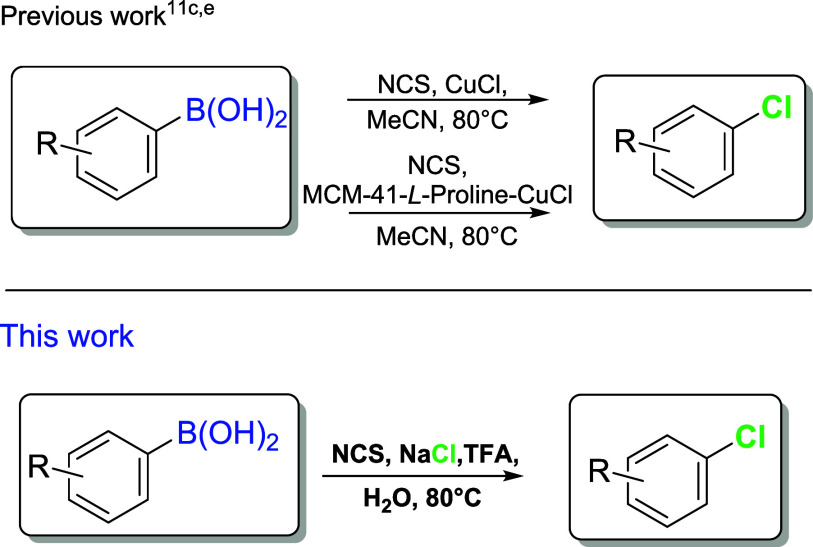
*Ipso*-Chlorination of Arylboronic Acids.

Water, a cheap, abundant, nontoxic, and nonflammable solvent, makes phase separation easy when used as a solvent because organic compounds can be directly extracted into an organic solvent by phase separation. Furthermore, by using the cheap and noncorrosive halide salts (NaBr and NaI), the corresponding bromo- and iodobenzenes have been synthesized with excellent yields and regioselectivity.

## Results and Discussion

The 4-methoxyphenylboronic acid was chosen as a model to optimize the reaction conditions, as reported in the subsequent table. Initially, we wanted to test organic solvent-free chlorination, which has been reported in the literature for the transformation of phenols into industrially relevant chlorinated compounds.^14^ Thus, the substrate was reacted with the NaCl/PTSA/NCS system in aqueous media under different reaction conditions (Table 1).

**Table 1:**
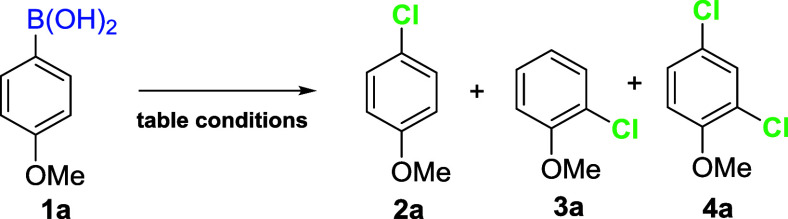
Optimization of Reaction Conditions

Entry^a^	acid	time (h)	*T* (°C)	**2** (%)	**3** (%)	**4**(%)	conv (%)
1	PTSA	3	r.t.	6	0	0	6
2	PTSA	1	65	48	7	0	55
3^b^	PTSA	1	65	32	16	3	51
4^c^	PTSA	1	65	41	1	0	44
5	PTSA	3	65	76	6	1	83
6	PTSA	6	65	90	2	3	95
7^d^	PTSA	1	65	83	4	10	100
8	PTSA	1	80	88	3	7	100
9^d^	PTSA	1	80	85	0	15	100
10	PTSA	3	80	89	2	8	100
11	CSA	1	80	82	0	5	87
12	TFA	1	80	88	0	0	88
13^e^	TFA	1	80	86	0	0	86
14	TFA	2	80	93	1	3	100
15		1	80	84	1	4	89
16^f^		1	80	77	1	2	80

a Conditions: **1** (0.15 mmol), NaCl (0.23 mmol), NCS (0.15 mmol), acid (0.15 mmol), water (0.5 mL).

b Without NaCl.

c With KF instead of NaCl.

d With 0.18 mmol of NCS.

e 0.1 equiv of acid.

f Reaction conducted in seawater.

At room temperature, only 6% of compound **2a** was obtained after 3 h (Table 1, entry 1), likely due to the low solubility of the boronic acid in water. Running the reaction at 65 °C resulted in higher conversions and yields, reaching 90% yield after 6 h (Table 1, entries 2, 5, 6). Running the reaction without salt at 65 °C for 1 h resulted in only a 32% yield (entry 3), whereas under the same conditions, in the presence of 1.5 equiv. of NaCl, a yield of 48% was obtained (Table 1, entry 4). The yield increased to 83% by changing the NCS equivalents from 1 to 1.2 at 65 °C for 1 h (Table 1, entry 7), even though the percentage of dichlorinated product increased from 0% to 10%. At 80 °C, the reaction showed faster kinetics without significant yield improvement (Table 1, entries 8–10). Reactions conducted at 80 °C for 1 h using 1.2 equiv. of NCS unexpectedly showed a yield decrease from 88% to 85%, while the percentage of dichlorinated product doubled from 7% to 15% (Table 1, entry 9). Changing the acid improved the reaction’s efficiency. Using TFA in catalytic amounts resulted in a modest decrease in yield compared to the use of stoichiometric amounts of TFA for 1 h of reaction (Table 1, entries 13 and 12, respectively) while using TFA for 2 h (Table 1, entry 14),gave the highest overall yield (93%) with quantitative conversion. Surprisingly, the reaction worked quite well even in the absence of acid (Table 1, entry 15), with an 84% yield and 89% conversion. All reactions led almost exclusively to monochlorination at the *ipso* position, with the percentage of dichlorinated product **4a** increasing with higher equivalents of NCS, reaching 15% when the reaction was conducted at 80 °C with 1.2 equiv. of NCS. An interesting finding emerged from entry 3: when the reaction was conducted without salt, a 2:1 *para*-/*ortho*-chlorinated mixture was observed. In all other cases, excellent regioselectivity was observed, especially for reactions run with TFA, CSA, or even in the absence of acid. By conducting the reaction in seawater without adding NaCl and without acid, a moderate yield of 77% of the desired product was achieved (Table 1, entry 16).

The optimized reaction conditions (Table 1, entry 14), in terms of time, temperature, and acid, were applied to substrates with various electron-withdrawing and electron-donating groups (Table 2).

**Table 2:**
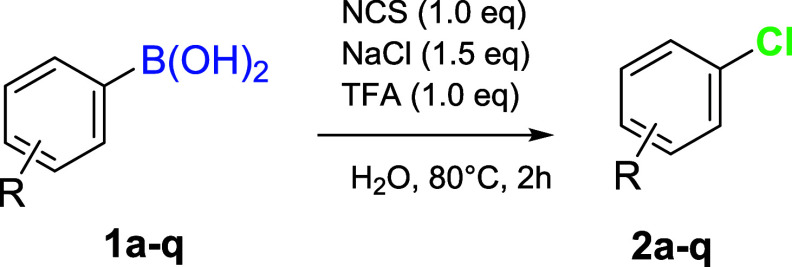

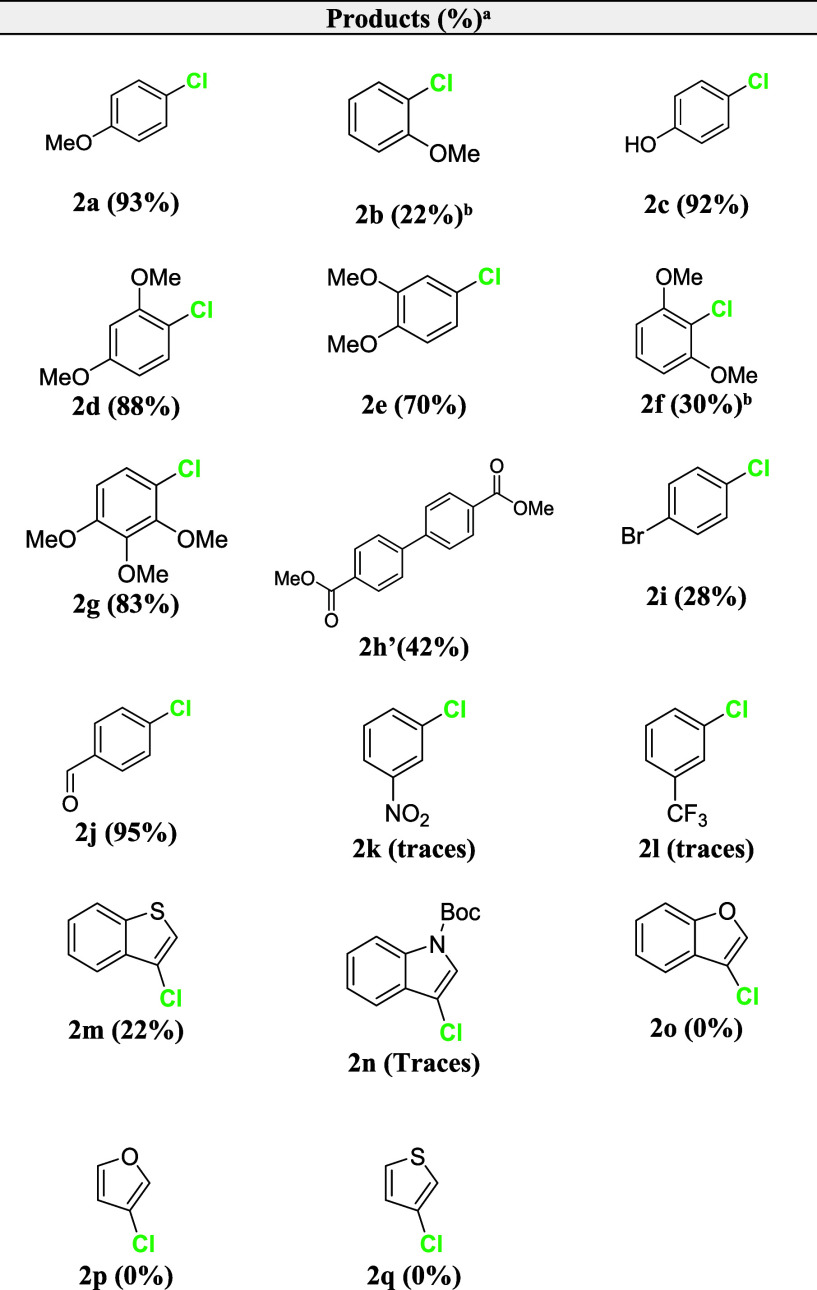
Substrate Scope for Chlorination of Phenylboronic Acids in Water

a Isolated yields.

b Isolated from a mixture of regioisomers.

With electron-donating substituents in the same position, generally high yields were obtained, ranging from 92% to 93% (**1c and 1a,** respectively). When polymethoxyphenyl boronic acids (**1d**–**g**) were used as substrates, yields ranged from good to excellent (for **1d, 1e, 1g**) and 30% for **1f**. *Ortho*-substituted systems with electron-donating groups (**1b** and **1f**) exhibited low regioselectivity and modest yields. The reaction worked well with electron-withdrawing substituents in the *para* position (**1h**–**j**), achieving reaction yields up to quantitative levels. With electron-withdrawing systems in the *meta* position, low yields were achieved (**1k** and **1l**). An unexpected result was obtained with substrate **1h**, as the reaction directly led to the formation of the biaryl compound **2h’**, through a transition-metal-free homocoupling process.

The reaction conducted on heterocycles (**1m**–**q**) resulted in a 22% yield for benzothiophene (**2m**) and only trace amounts of the desired product for the protected indole (**2n**), while leading to substrate degradation when performed on furans and benzofurans (**2pand 2o,** respectively).

Given the encouraging results, we further investigated this potential *ipso* halogenation methodology of substituted boronic acids for bromination and iodination (Table 3). This was accomplished by maintaining the same reaction conditions and using the appropriate halogenated salt (NaBr or NaI). Bromination and iodination of p-methoxyphenylboronic acid **1a** (Table 3, entry 1) resulted in excellent yields of 89% and 82%, respectively.

**Table 3:**
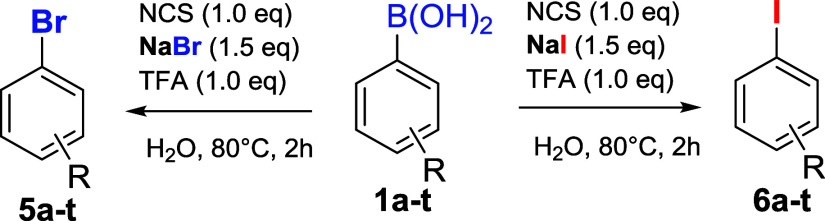

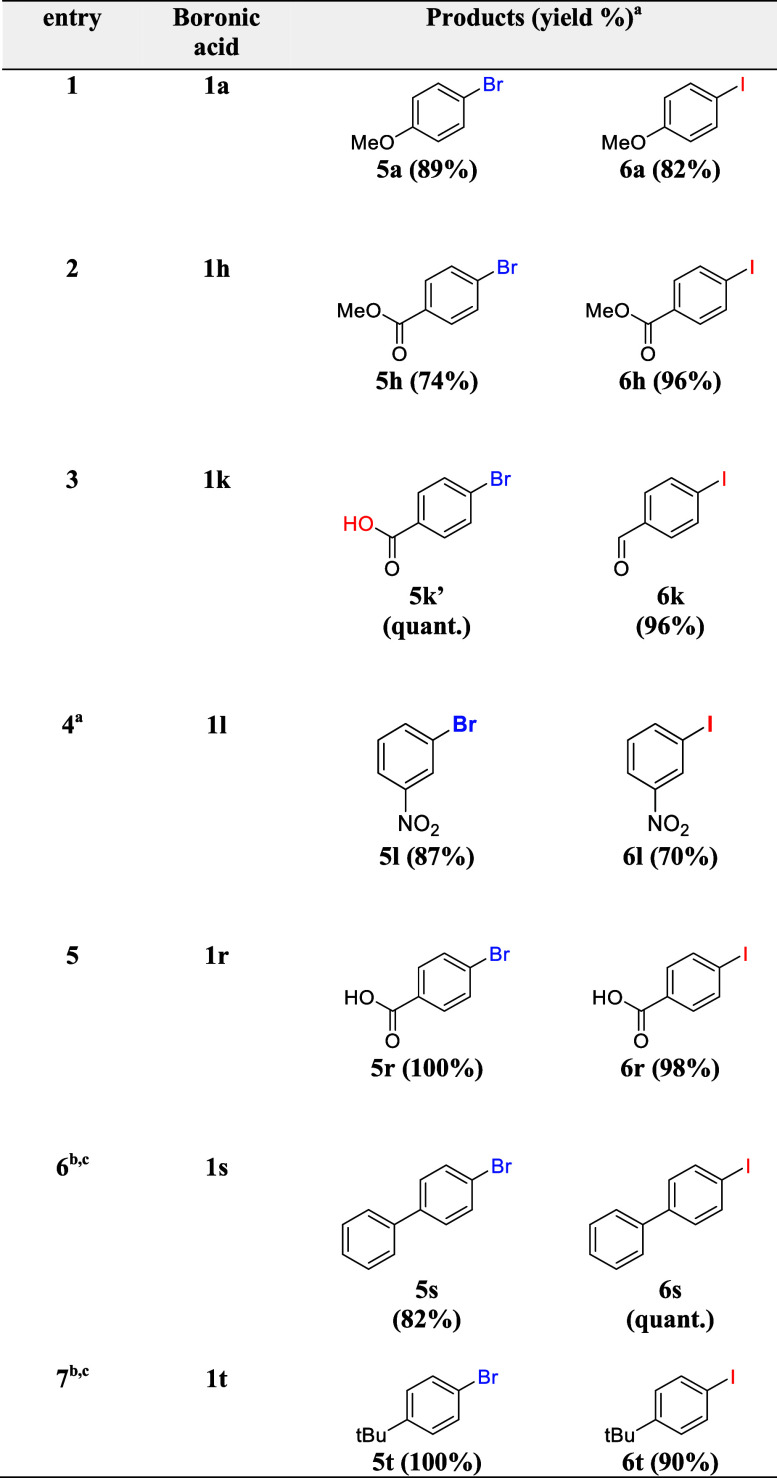
Substrate Scope for Bromination and Iodination of Phenylboronic Acids in Water

a Isolated yield.

b Run for 16 h.

c Chlorination gave traces of products.

For the bromination, a remarkable result was obtained with *p-*formylphenylboronic acid **1k** (Table 3, entry 3**)**, yielding the corresponding *p*-Br-benzoic acid **5k’** quantitatively. A similar outcome was reported in the literature as a side reaction during the bromination of benzaldehyde with the NaBrO_3_/H_3_PO_4_ system, where *meta*-bromination and simultaneous oxidation to benzoic acid occur, resulting in a mixture of 3-Br-benzaldehyde/3-Br-benzoic acid in a 7:2 ratio.^15^ The iodination of the same substrate **1k**, however, led to the formation of 4-I-benzaldehyde **6k** in 96% yield. This is particularly significant as product **6k** is commercially more valuable compared to the corresponding boronic acid. The bromination of *m*-nitrophenylboronic acid **1l** led to the formation of the desired *ipso*-bromination product with an 87% yield, while iodination resulted in the desired product **6l** with a 70% yield.

The bromination of *p*-phenyl phenylboronic acid **1s** yielded the expected product **5s** in 82% yield, whereas the analogous iodination provided a quantitative yield of compound **6s** (Table 3, entry 6). Finally, the halogenation of the boronic acid with a weakly electron-donating substituent, such as *t*-Bu **1t,** yielded the brominated product **5t** quantitatively and the iodinated product **6t** with a 90% yield (Table 3, entry 7).

Concerning the reaction mechanism, it could not involve an electrophilic aromatic substitution as in our previous work on *ipso*-formylation of phenylboronic acids,^16^ but probably proceeds via a direct migration of the aryl moiety (activated or deactivated arenes) from boron to halogen. First, the acid activates NCS to generate the corresponding *X*Cl species (species A in Scheme 2), which could directly participate in the formation of the corresponding aryl halide through the formation of a four-membered intermediate (via A, Scheme 2). Alternatively, *X*Cl could generate the corresponding NXS (species B in Scheme 2), NBS, or NIS in the presence of Na*X* (NaBr, NaI).^17^ In this case, the conversion of boron to a borate species via the addition of an external nucleophile such as Cl^–^, added or generated in situ, may lead to an intermolecular transfer of the aryl ring to the electrophilic halogen (via B, Scheme 2). Indeed, the *ipso*-iodination of *p*-methoxyphenylboronic acid (**1a**) with NIS in water yielded the desired product **2a** with yields comparable to those discussed in Table 3, suggesting that the formation of NXS may be involved in the reaction mechanism.

**Scheme 2:**
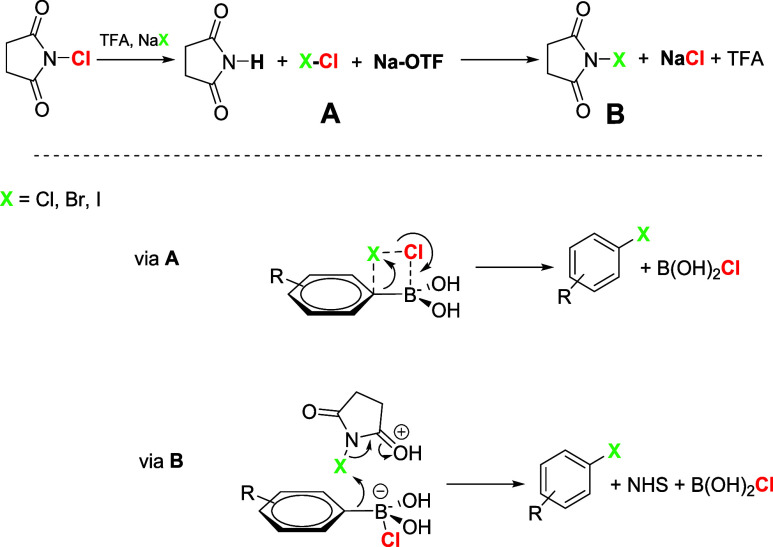
Proposed Mechanism

These considerations stem from the need to understand why additional NaCl is fundamental for enhancing the yield of the chlorinated aryl compounds **2a**–**r** and to explain the similar reactivity of electron-rich or electron-deficient rings of the corresponding phenylboronic acids. Moreover, in the absence of NaCl, the reaction on **1a** exhibits poor yields and low regioselectivity, resulting in a 2:1 *para*-/*ortho*-chlorinated mixture (entry 3, Table 1). In conclusion, a new method for the *ipso-*chlorination of arylboronic acids in water, without the use of a transition metal catalyst, was developed, which operates efficiently even on nonactivated or activated systems. Furthermore, by using the NCS/NaX system, the corresponding bromo- and iodo-arenes were synthesized in water with excellent yields and regioselectivity.

## Supplementary Material



## Notes

Experimental procedures, analytical data for all new compounds, NMR spectra (PDF)
