# Supplementary material for: NCS-Mediated Ipso-Halogenation of Arylboronic Acids in Water Using Sodium Halides
Source: ACS Omega. 2025 Jun 25;10(26):27856–60. doi: 10.1021/acsomega.5c00755 (PMC12242620; doi:10.1021/acsomega.5c00755)
Supplement: Supplementary file 1 [file ao5c00755_si_001.pdf]

SUPPORTING INFORMATION

NCS Mediated *Ipso*-halogenation of Arylboronic Acids in Water Using Sodium Halides

Alessandro Santarsiere,[a*] Pierantonio Galgano,[a] Maria Funicello,[a] Paolo Lupattelli,[b] Lucia Chiummiento.[a*]

[a]*Department of Basic and Applied Sciences, University of Basilicata Via dell'Ateneo lucano 10, 85100 Potenza (Italy)*

[b] *Department of Chemistry, University "La Sapienza" of Roma, Piazzale A. Moro 5, 00185 Roma (Italy)*

Table of Contents

1. **General Procedures S2**
2. **General procedure for *ipso*-chlorination S2**
3. **General procedure for *ipso*-bromination S4**
4. **General procedure for *ipso*-iodination S5**
5. **NMR Spectra S7**
6. **References S31**

1. General procedures

All reagents were supplied by Sigma-Aldrich, TCI and AlfaAesar companies and were used without further purification unless otherwise stated. All reactions were carried out in oven-dried glassware under an argon atmosphere unless otherwise noted. Flash chromatography was performed using 60–200 mesh silica gel. ^{1}H NMR spectra were recorded on Varian 400 MHz and Varian 500 MHz spectrometer at room temperature with $CDCl_3$ as the solvent unless otherwise noted. Chemical shifts are reported in parts per million relative and referenced internally to the residual solvent resonances: 1H NMR spectra to $CDCl_3$ at δ 7.26. Data for ^{1}H NMR are reported as follows: chemical shift, multiplicity (s = singlet, d = doublet, t = triplet, q = quartet, m = multiplet), coupling constants (in Hertz) and integration.

2. General procedure for ipso-chlorination:

NCS (0.15 mmol), NaCl (0.23 mmol) and TFA (0.15 mmol) were added to a solution of the substrate (0.15 mmol) in distilled water (1 mL). The resulting solution was stirred at 80° C for 1 h. The reaction mixture was then diluted with AcOEt (1 mL) and washed with water. The organic layer was dried with Na_2SO_4, filtered, and concentrated.

1-chloro-4-methoxybenzene (2a)

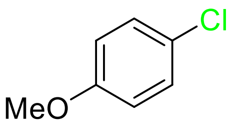

2a

Compound **2a** was obtained using the general procedure for *ipso*-chlorination with 93% yield. ^{1}H NMR (400 MHz, $CDCl_3$) δ = 7.24 (d, *J* = 9.0 Hz, 2H), 6.83 (d, *J* = 9.0 Hz, 2H), 3.79 (s, 3H) ppm.[1]

1-chloro-2-methoxybenzene (2b)

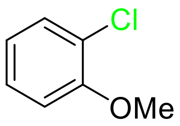

2b

Compound **2b** was obtained using the general procedure for *ipso*-chlorination and it was purified by column chromatography on silica gel (petroleum ether/AcOEt 8:2) affording **2b** (22%). ^{1}H NMR (400 MHz, $CDCl_3$) 7.36 (d, *J* = 8.0 Hz, 1H), 7.23 (t, *J* = 7.4 Hz, 1H), 6.93 (d, *J* = 8.5 Hz, 1H), 6.90 (t, *J* = 7.2 Hz, 1H), 3.91 (s, 3H) ppm.[2]

4-chlorophenol (2c)

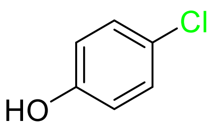

2c

Compound **2c** was obtained using the general procedure for *ipso*-chlorination and it was purified by column chromatography on silica gel (petroleum ether /AcOEt 8:2) affording **2c** (92%). ^{1}H NMR (500 MHz, $CDCl_3$) δ = 7.19 (d, *J* = 8.8 Hz, 2H), 6.78 (d, 8.8 Hz, 2H) ppm.[3]

1-chloro-2,4-dimethoxybenzene (2d)

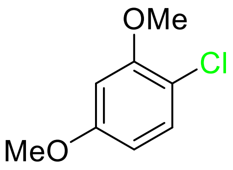

2d

Compound **2d** was obtained using the general procedure for *ipso*-chlorination and it was purified by column chromatography on silica gel (petroleum ether /AcOEt 8:2) affording **2d** (88%) ^{1}H NMR (400 MHz, $CDCl_3$) δ = 7.24 (d, *J* = 8.7 Hz, 1H), 6.50 (d, 2.5 Hz, 1H), 6.43 (dd, *J'* = 8.7 Hz, *J''* = 2.6 Hz, 1H), 3.87 (s, 3H), 3.79 (s, 3H) ppm.[4]

4-chloro-1,2-dimethoxybenzene (2e)

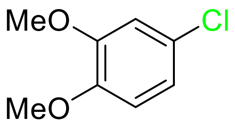

2e

Compound **2e** was obtained using the general procedure for *ipso*-chlorination and it was purified by column chromatography on silica gel (petroleum ether/AcOEt 8:2) affording **2e** (70%) ^{1}H NMR (400 MHz, $CDCl_3$) δ = 6.88 (dd, *J'* = 8.5 Hz, *J''* = 2.2 Hz, 1H), 6.85 (d, *J* = 2.1 Hz, 1H), 6.77 (d, *J* = 8.4 Hz, 1H), 3.86 (s, 3H), 3.87 (s, 3H) ppm.[4]

2-chloro-1,3-dimethoxybenzene (2f)

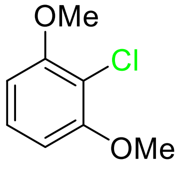

2f

Compound **2f** was obtained using the general procedure for *ipso*-chlorination and it was purified by column chromatography on silica gel (petroleum ether/AcOEt 8:2) affording **2f** (30%) ^{1}H NMR (400 MHz, $CDCl_3$) δ = 6.88 (dd, *J'* = 8.5 Hz, *J''* = 2.2 Hz, 1H), 6.85 (d, *J* =2.1 Hz, 1H), 6.77 (d, *J* = 8.4 Hz, 1H), 3.86 (s, 3H), 3.87 (s, 3H) ppm.[5]

1-chloro-2,3,4-trimethoxybenzene (2g)

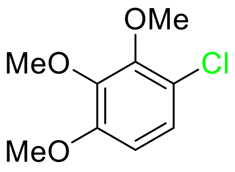

2g

Compound **2g** was obtained using the general procedure for *ipso*-chlorination and it was purified by column chromatography on silica gel (petroleum ether/AcOEt 8:2) affording **2g** (83%) ^{1}H NMR (400 MHz, $CDCl_3$) δ = 7.05 (d, *J* =8.9 Hz, 1H), 6.61 (d, *J* =8.9 Hz, 1H), 3.91 (s, 3H), 3.88 (s, 3H), 3.85 (s, 3H) ppm.[6]

1-chloro-2,3,4-trimethoxybenzene (2h')

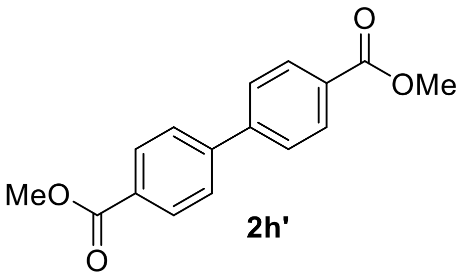

Compound **2h'** was obtained using the general procedure for *ipso*-chlorination and it was purified by column chromatography on silica gel (petroleum ether/AcOEt 8:2) affording **2h'** (42%) ^{1}H NMR (400 MHz, $CDCl_3$) δ = 8.13 (d, *J* =7.8 Hz, 1H), 7.70 (d, *J* =7.8 Hz, 1H), 3.95 (s, 3H) ppm.[7]

1-bromo-4-chlorobenzene (2i) and **1,4-dibromobenzene (2i')**

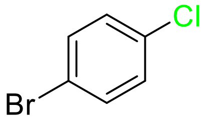

2i

+

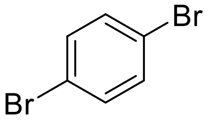

2i'

Compound **2i** and **2i'** were obtained using the general procedure for *ipso*-chlorination and it was purified by column chromatography on silica gel (petroleum ether/AcOEt 8:2) affording **2i** (28%) ^{1}H NMR (500 MHz, $CDCl_3$) δ = 7.42 (d, *J* = 8.5 Hz, 2H), 7.21 (d, *J* =8.5 Hz, 2H) ppm[4] and compound **2i'** ^{1}H NMR (500 MHz, $CDCl_3$) δ = 7.36 (s, 4H) ppm.[9]

4-chlorobenzaldehyde (2j)

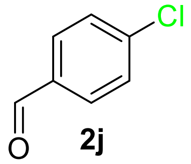

Compound **2j** was obtained using the general procedure for *ipso*-chlorination and it was purified by column chromatography on silica gel (petroleum ether/AcOEt 8:2) affording **2j** (95%). ^{1}H NMR (400 MHz, $CDCl_3$) δ = 9.99 (s, 1H), 7.82 (d, *J* = 8.1 Hz, 2H), 7.52 (d, *J* = 8.1 Hz, 2H) ppm.[10]

3-chlorobenzothiophene (2m)

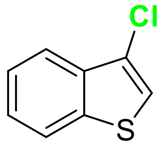

2m

Compound **2m** was obtained using the general procedure for *ipso*-chlorination and it was purified by column chromatography on silica gel (petroleum ether/AcOEt 8:2) affording **2m** (22%). ^{1}H NMR (400 MHz, $CDCl_3$) δ = 7.83-7.87 (m, 2H), 7.35-7.49 (m, 2H), 7.32 (s,1H) ppm.[11]

3. **General procedure for ipso-bromination**:

NCS (0.15 mmol), NaBr (0.23 mmol) and TFA (0.15 mmol) were added to a solution of the substrate (0.15 mmol) in distilled water (1 mL). The resulting solution was stirred at 80° C for 1 h. The reaction mixture was then diluted with Et_2O (1 mL) and washed with brine. The organic layer was dried with Na_2SO_4, filtered, and concentrated.

1-bromo-4-methoxybenzene (5a)

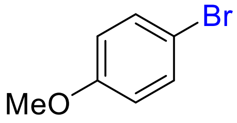

5a

Compound **5a** was obtained using the general procedure for *ipso*-bromination and it was purified by column chromatography on silica gel (petroleum ether/AcOEt 8:2) affording **5a** (89%) ^{1}H NMR (400 MHz, $CDCl_3$) δ = 7.38 (d, *J* = 8.8 Hz, 2H), 6.78 (d, *J* = 8.8 Hz, 2H), 3.78 (s, 3H) ppm.[12]

methyl 4-bromobenzoate (5h)

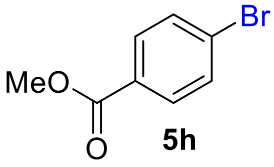

5h

Compound **5h** was obtained using the general procedure for *ipso*-bromination and it was purified by column chromatography on silica gel (petroleum ether/AcOEt 8:2) affording **5h** (74%). ^{1}H NMR (400 MHz, $CDCl_3$) δ = 7.89 (d, *J* =8.6 Hz, 2H), 7.57 (d, *J* = 8.6 Hz, 2H) ppm.[13]

4-bromobenzoic acid (5k')

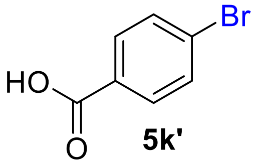

5k'

Compound **5k'** was obtained using the general procedure for *ipso*-bromination of **2k** affording **5k'** with quantitative yield. ^{1}H NMR (400 MHz, $CDCl_3$) δ = 7.94 (d, *J* =8.8 Hz, 2H), 7.61 (d, *J* = 8.8 Hz, 2H) ppm.[8]

1-bromo-3-nitrobenzene (5l)

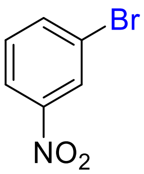

5l

Compound **5l** was obtained using the general procedure for *ipso*-bromination by using 70mg (0.42 mmol) of the boronic acid **1l** and it was purified by column chromatography on silica gel (petroleum ether/AcOEt 8:2) affording **5l** (87%). ^{1}H NMR (400MHz, $CDCl_3$) δ = 8.36 (bs, 1H), 8.16 (m, 1H), 7.83 (m, 1H), 7.44 (t, *J* = 8.0 Hz, 1H) ppm.[14]

4-bromo-1,1'-biphenyl (5s)

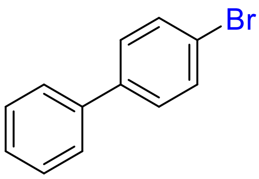

5s

Compound **5s** was obtained using the general procedure for *ipso*-bromination and it was purified by column chromatography on silica gel (petroleum ether/AcOEt 8:2) affording **5s** (82%). ^{1}H NMR (400 MHz, $CDCl_3$) δ = 7.56-7.58 (m, 4H), 7.38-7.47 (m, 5H) ppm.[15]

1-bromo-4-(tert-butyl)benzene (5t)

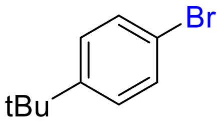

5t

Compound **5t** was obtained using the general procedure for *ipso*-bromination affording **5t** with quantitative yield. ^{1}H NMR (400 MHz, $CDCl_3$) δ = 7.41 (d, J = 8.4 Hz, 2H), 7.26 (d, J = 8.4 Hz, 2H), 1.30 (s, 9H) ppm.[16]

4. **General procedure for ipso-iodination**:

NCS (0.15 mmol), NaI (0.23 mmol) and TFA (0.15 mmol) were added to a solution of the substrate (0.15 mmol) in distilled water (1 mL). The resulting solution was stirred at 80° C for 1 h. The reaction mixture was then diluted with AcOEt (1 mL) and washed with water. The organic layer was dried with Na_2SO_4, filtered, and concentrated.

1-Iodo-4-methoxybenzene (6a)

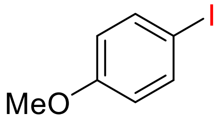

6a

Compound **6a** was obtained using the general procedure for *ipso*-iodination and it was purified by column chromatography on silica gel (petroleum ether/AcOEt 8:2) affording **6a** (82%) ^{1}H NMR (400 MHz, $CDCl_3$) δ = 7.55 (d, J = 8.8 Hz, 2H), 6.67 (d, J = 8.8 Hz, 2H), 3.78 (s, 3H) ppm.[17]

methyl 4-iodobenzoate (6h)

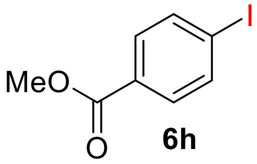

6h

Compound **6h** was obtained using the general procedure for *ipso*-iodination and it was purified by column chromatography on silica gel (petroleum ether/AcOEt 8:2) affording **6h** (96%). ^{1}H NMR (400 MHz, $CDCl_3$) δ = 7.80 (d, J = 8.6 Hz, 2H), 7.74 (d, J = 8.6 Hz, 2H), 3.81 (s, 3H) ppm.[13]

4-iodobenzaldehyde (6k)

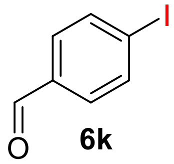

6k

Compound **6k** was obtained using the general procedure for *ipso*-iodination affording **6k** (96%). ^{1}H NMR (400 MHz, $CDCl_3$) δ = 9.96 (s, 1H), 7.92 (d, J = 8.2 Hz, 2H), 7.59 (d, J = 8.3 Hz, 2H) ppm.[19]

1-iodo-3-nitrobenzene (6l)

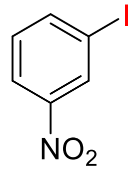

6l

Compound **6l** was obtained using the general procedure for *ipso*-iodination by using 50mg (0.3 mmol) of the boronic acid **1l** and it was purified by column chromatography on silica gel (petroleum ether /AcOEt 8:2) affording **6i** (70%). ^{1}H NMR (400 MHz, $CDCl_3$) δ = 8.56 (s, 1 H), 8.21 (d, *J*= 8.2 Hz, 1 H), 8.03 (d, *J*= 8.2 Hz, 1 H), 7.30 (t, *J*=8.1 Hz, 1 H) ppm.[20]

4-iodobenzoic acid (6r)

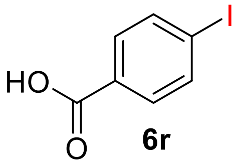

6r

Compound **6r** was obtained using the general procedure for *ipso*-iodination and it was purified by column chromatography on silica gel (petroleum ether/AcOEt 8:2) affording **6r** (98%). ^{1}H NMR (400 MHz, $CDCl_3$) δ =7.86-7.78 (m, 4H) ppm.[18]

4-iodo-1,1'-biphenyl (6s)

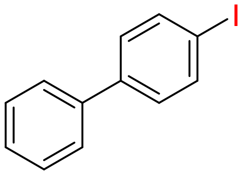

6s

Compound **6s** was obtained using the general procedure for *ipso*-iodination affording **6s** with quantitative yield. ^{1}H NMR (400 MHz, $CDCl_3$) δ = 7.76-7.78 (m, 2H), 7.57-7.54 (m, 2H), 7.47-7.42 (m, 2H), 7.40-7.33 (m, 3H) ppm.[21]

1-(tert-butyl)-4-iodobenzene (6t)

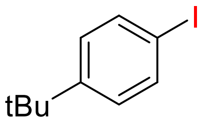

6t

Compound **6t** was obtained using the general procedure for *ipso*-iodination and it was purified by column chromatography on silica gel (petroleum ether/AcOEt 8:2) affording **6t** (90%). ^{1}H NMR (400 MHz, $CDCl_3$) δ = 7.62 (d, *J* =8.5 Hz, 2H), 7.15 (d, *J* = 8.5 Hz, 2H), 1.29 (s, 9H) ppm.[22]

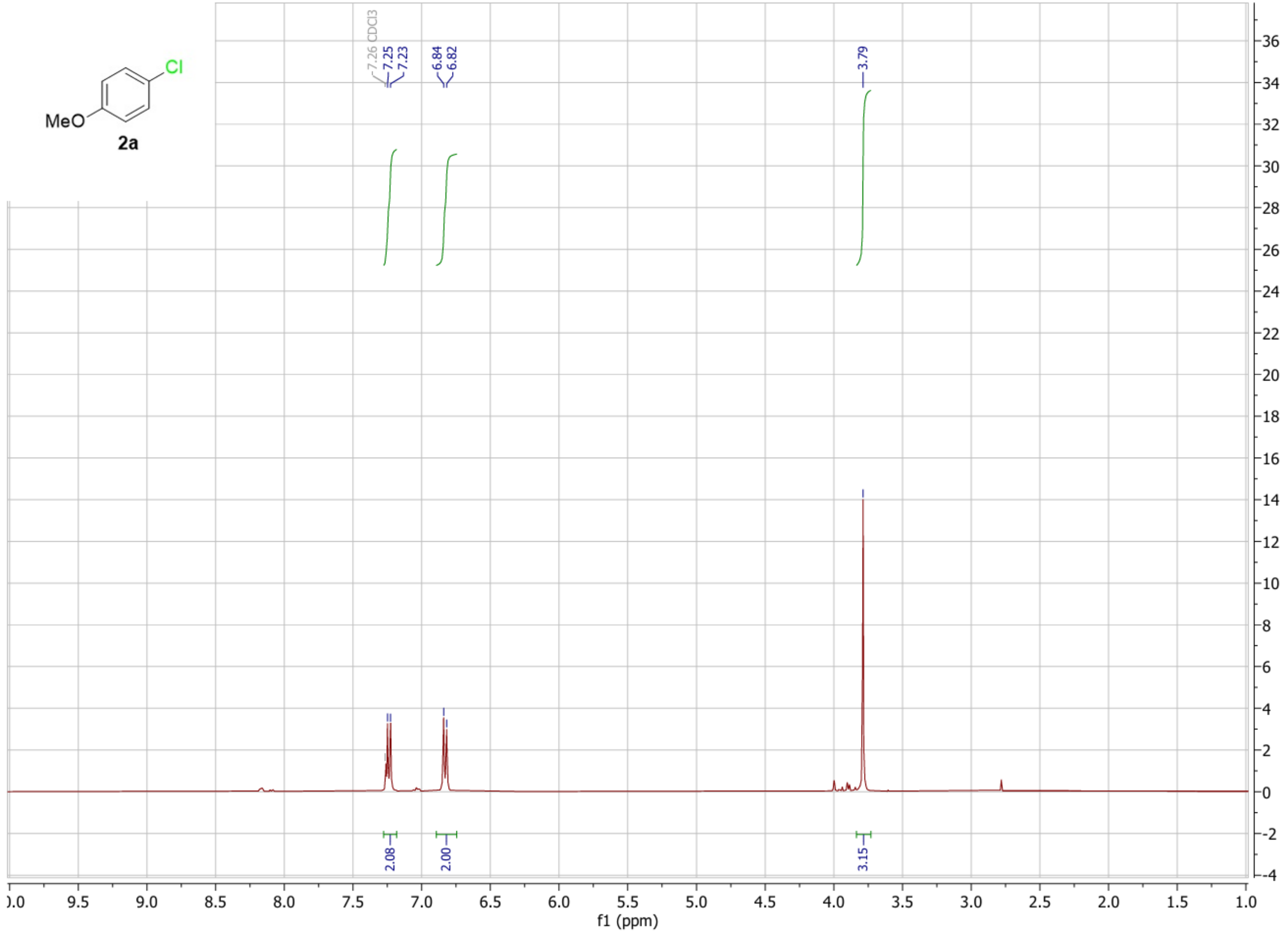

Figure S1. ^{1}H NMR (400 MHz, $CDCl_3$) of **2a**

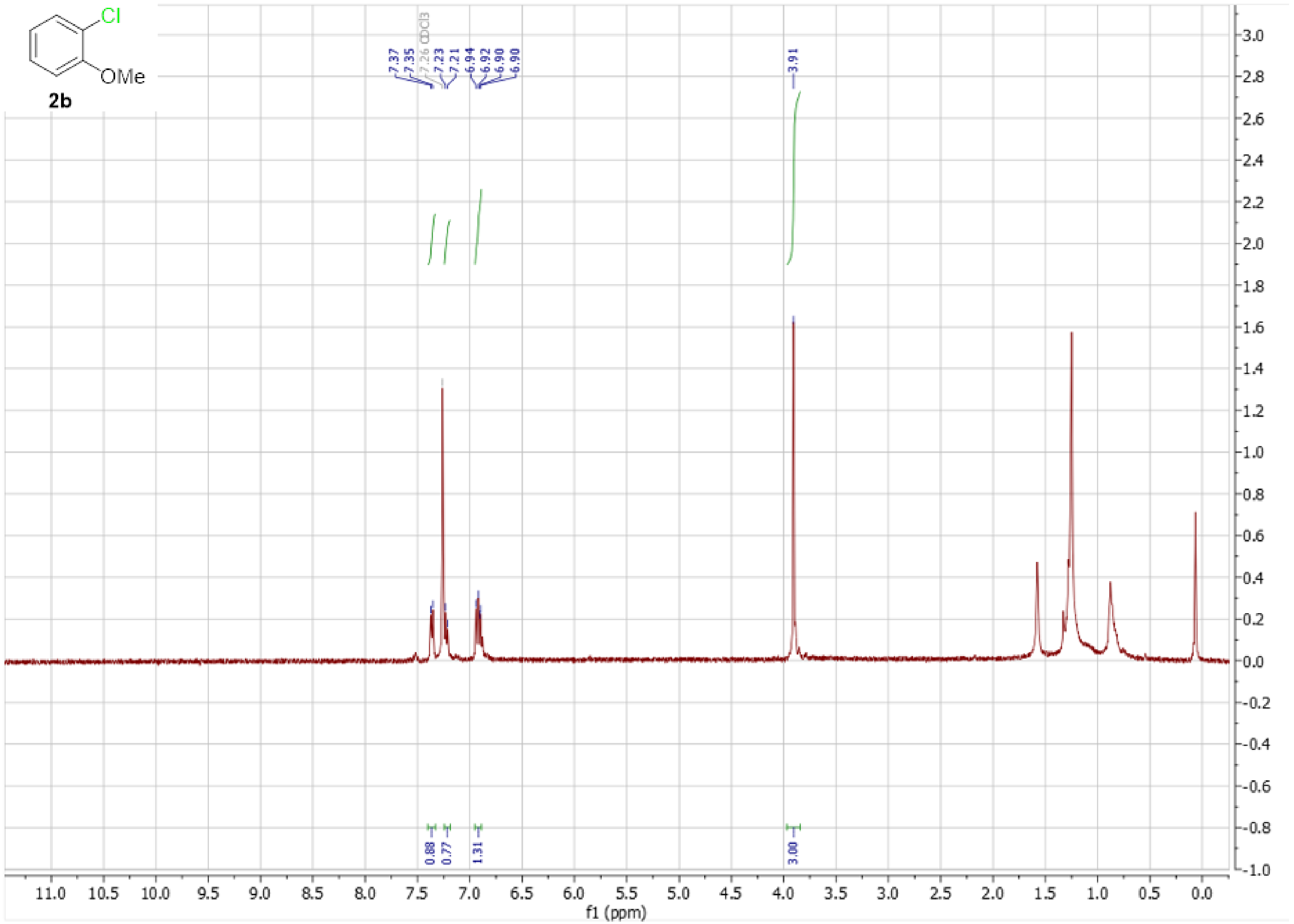

Figure S2. ^{1}H NMR (400 MHz, $CDCl_3$) of **2b**

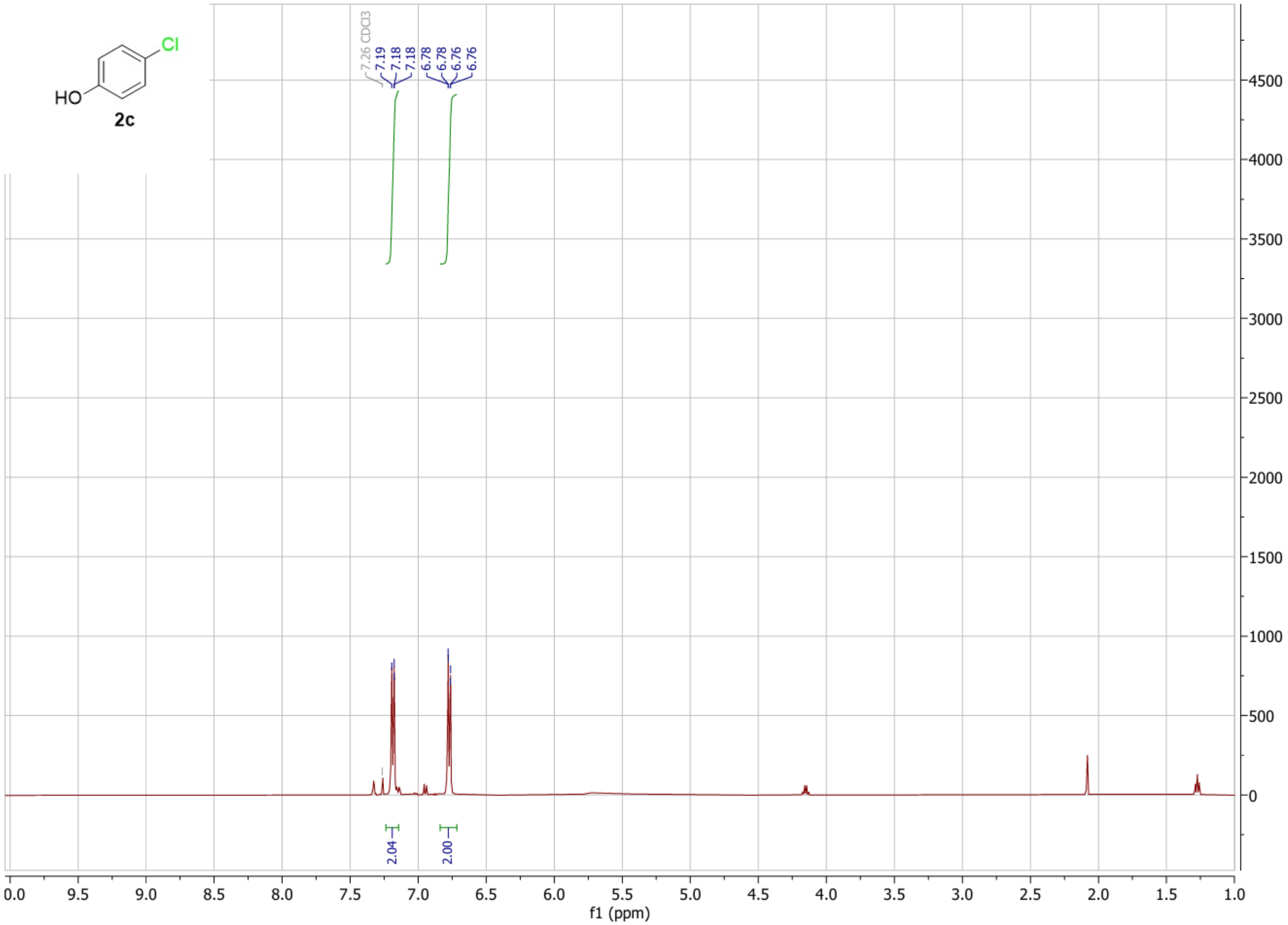

Figure S3. ^{1}H NMR (400 MHz, $CDCl_3$) of **2c**

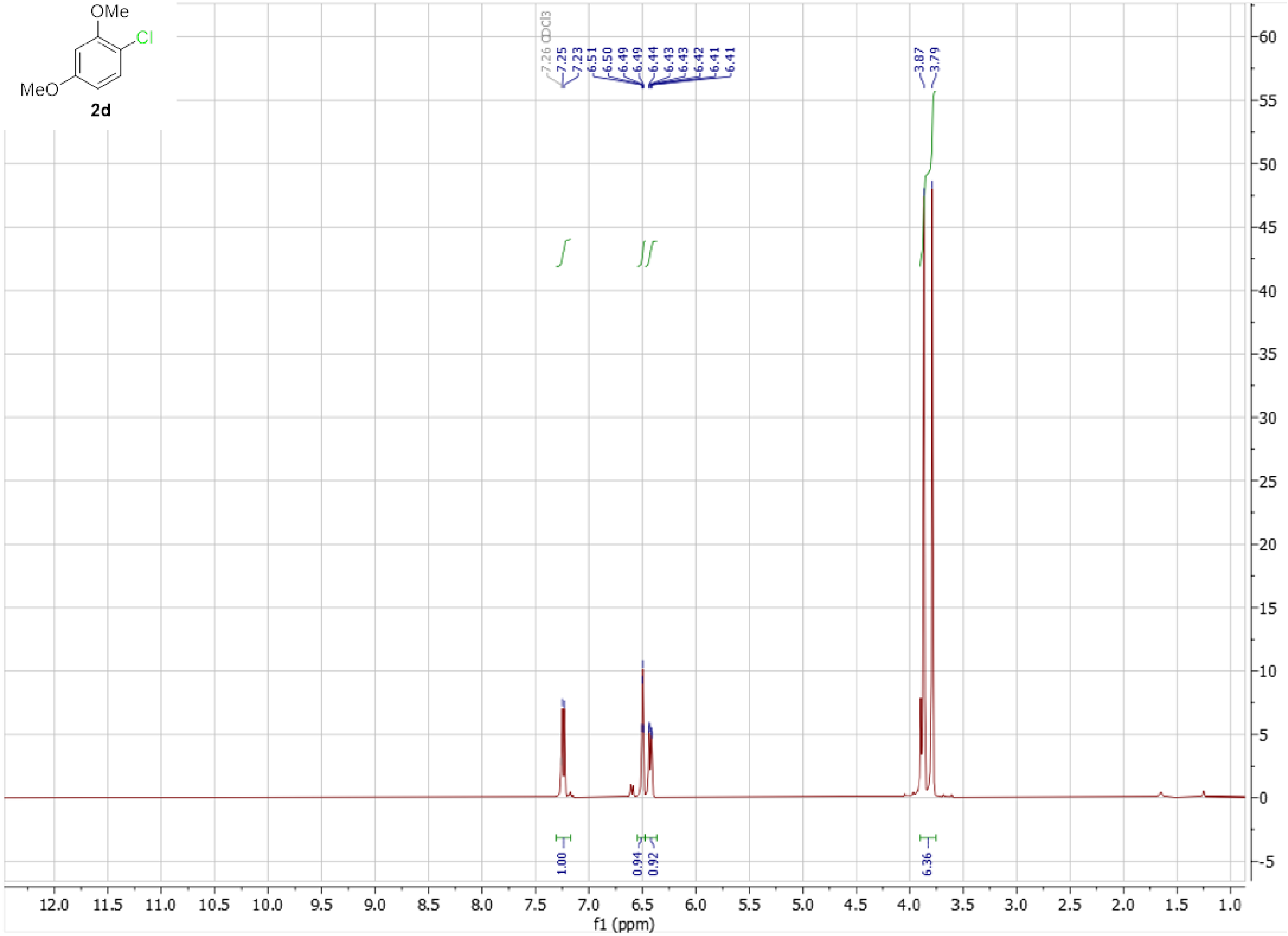

Figure S4. ^{1}H NMR (400 MHz, $CDCl_3$) of **2d**

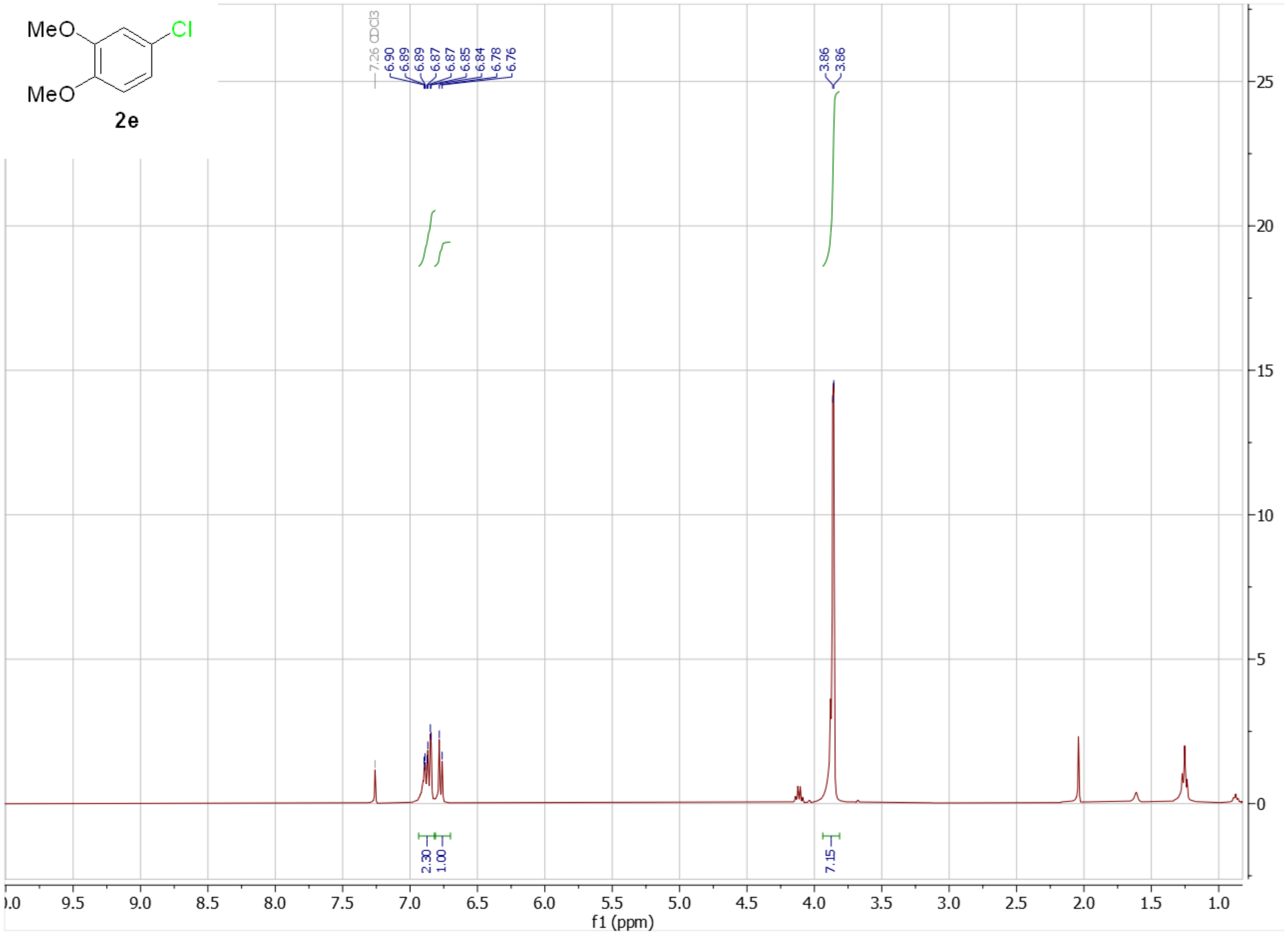

Figure S5. ^{1}H NMR (400 MHz, $CDCl_3$) of **2e**

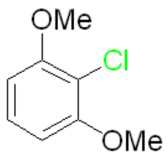

2f

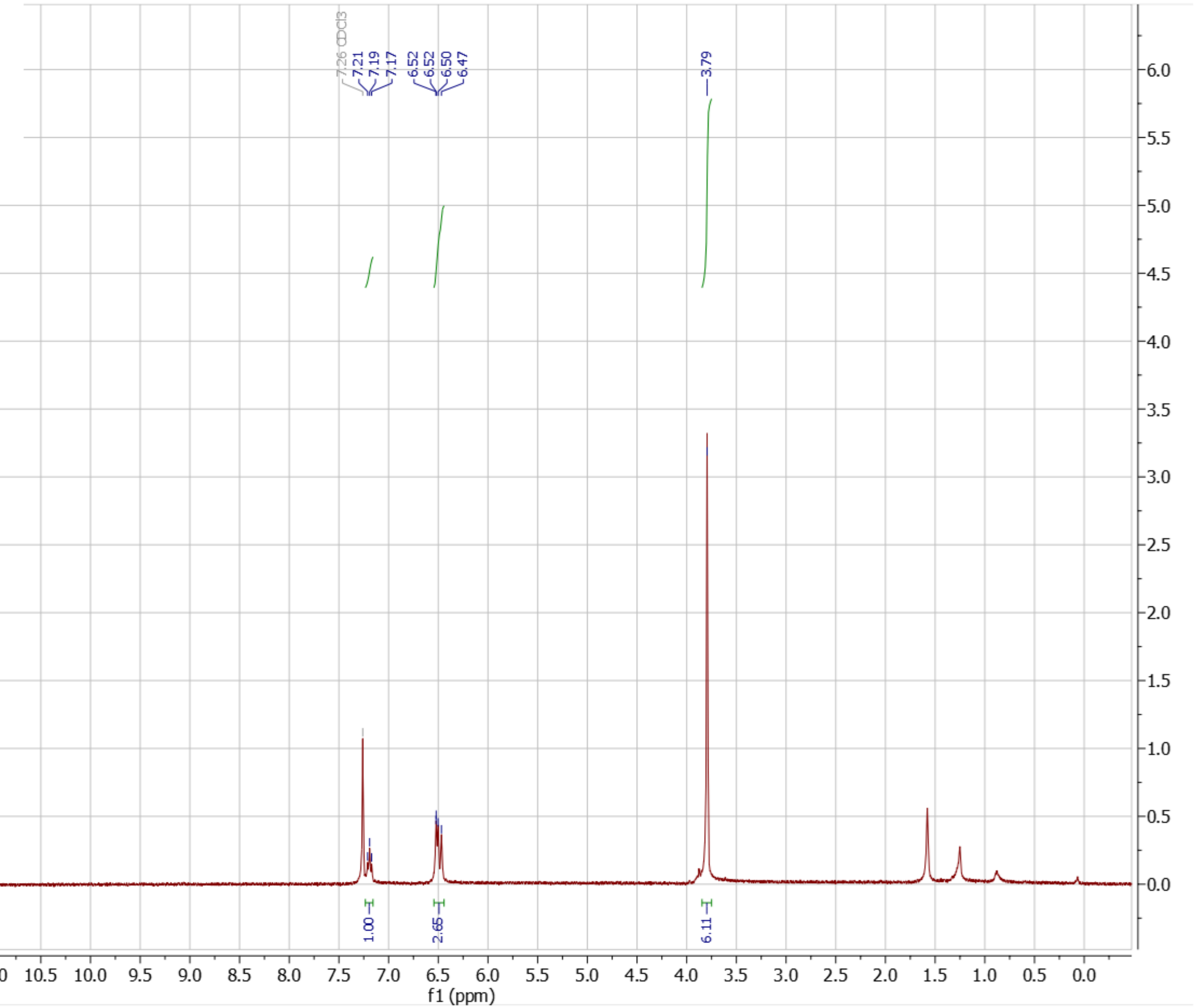

Figure S6. ^{1}H NMR (400 MHz, $CDCl_3$) of **2f**

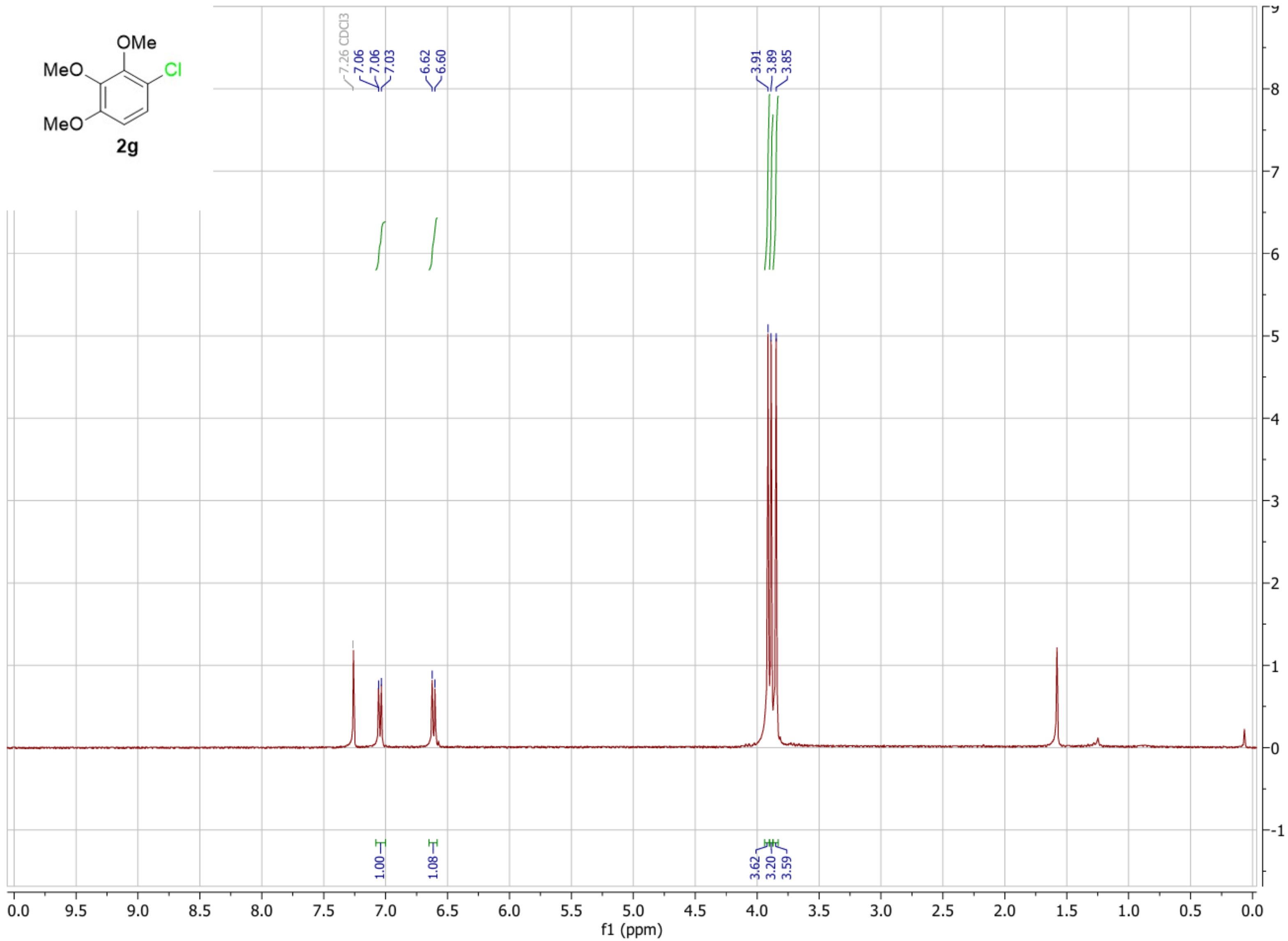

Figure S7. ^{1}H NMR (400 MHz, $CDCl_3$) of **2g**

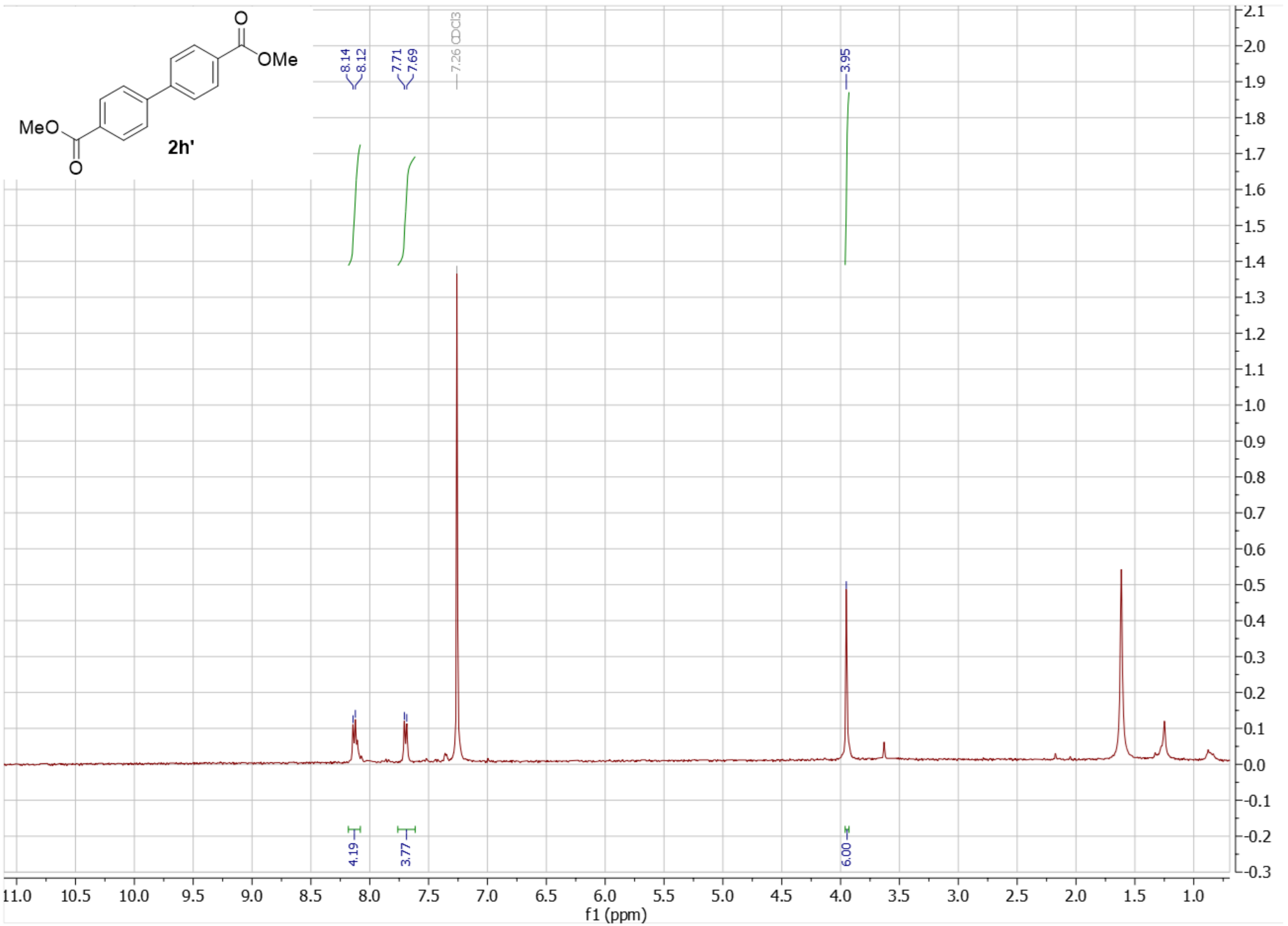

Figure S8. ^{1}H NMR (400 MHz, $CDCl_3$) of **2h’**

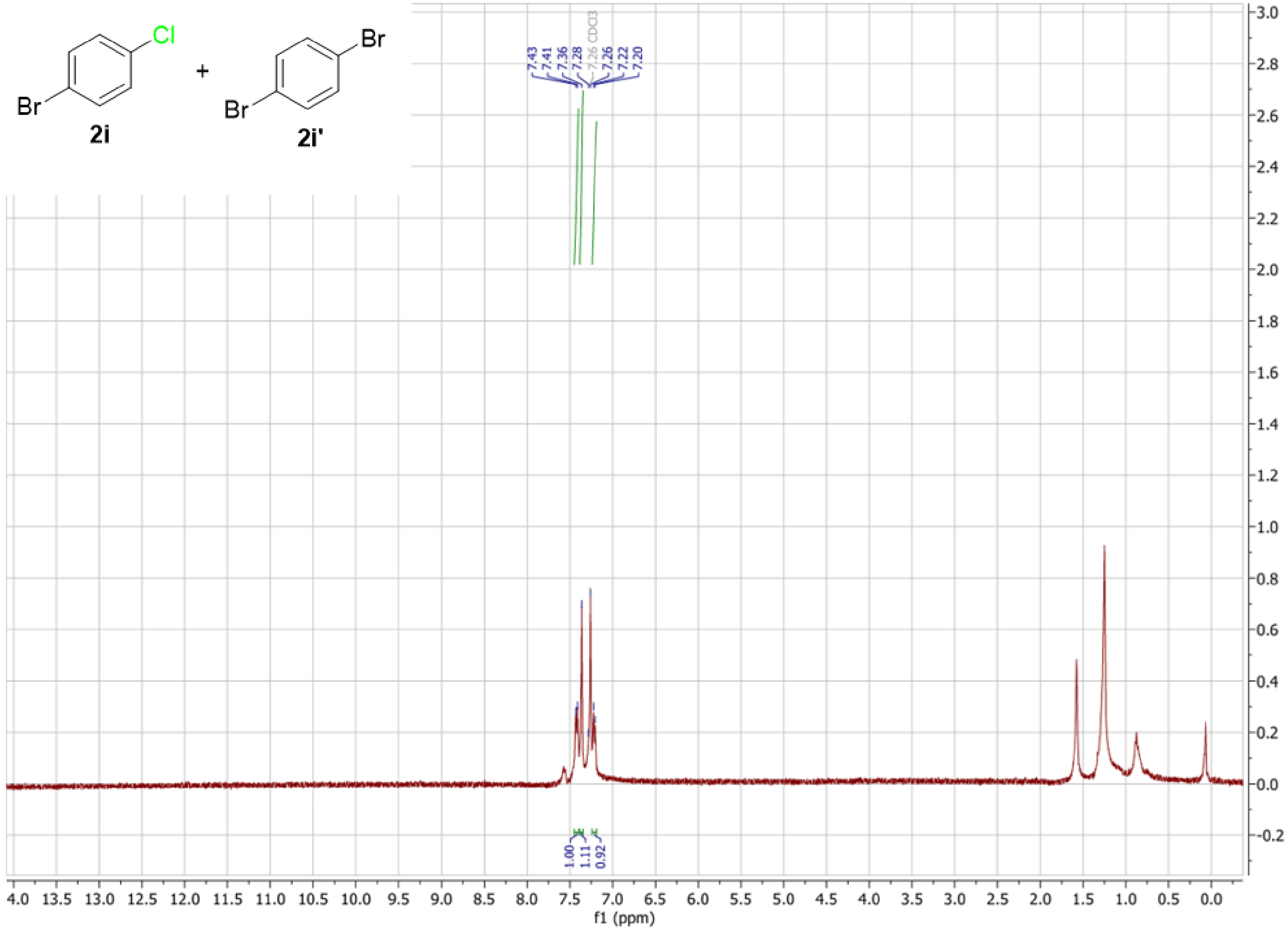

Figure S9. ^{1}H NMR (400 MHz, $CDCl_3$) of **2i** and **2i'**

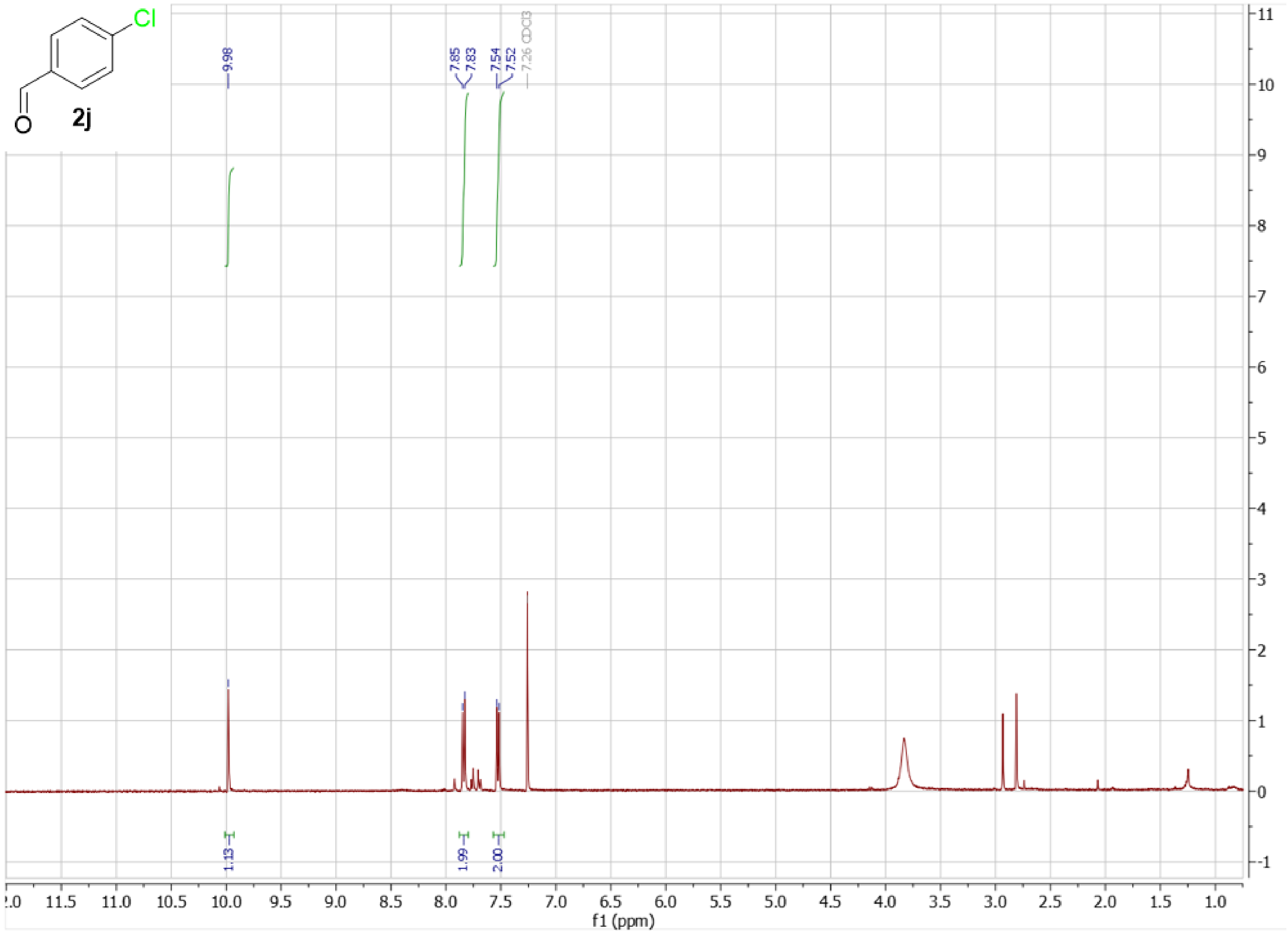

Figure S10. ^{1}H NMR (400 MHz, $CDCl_3$) of **2j**

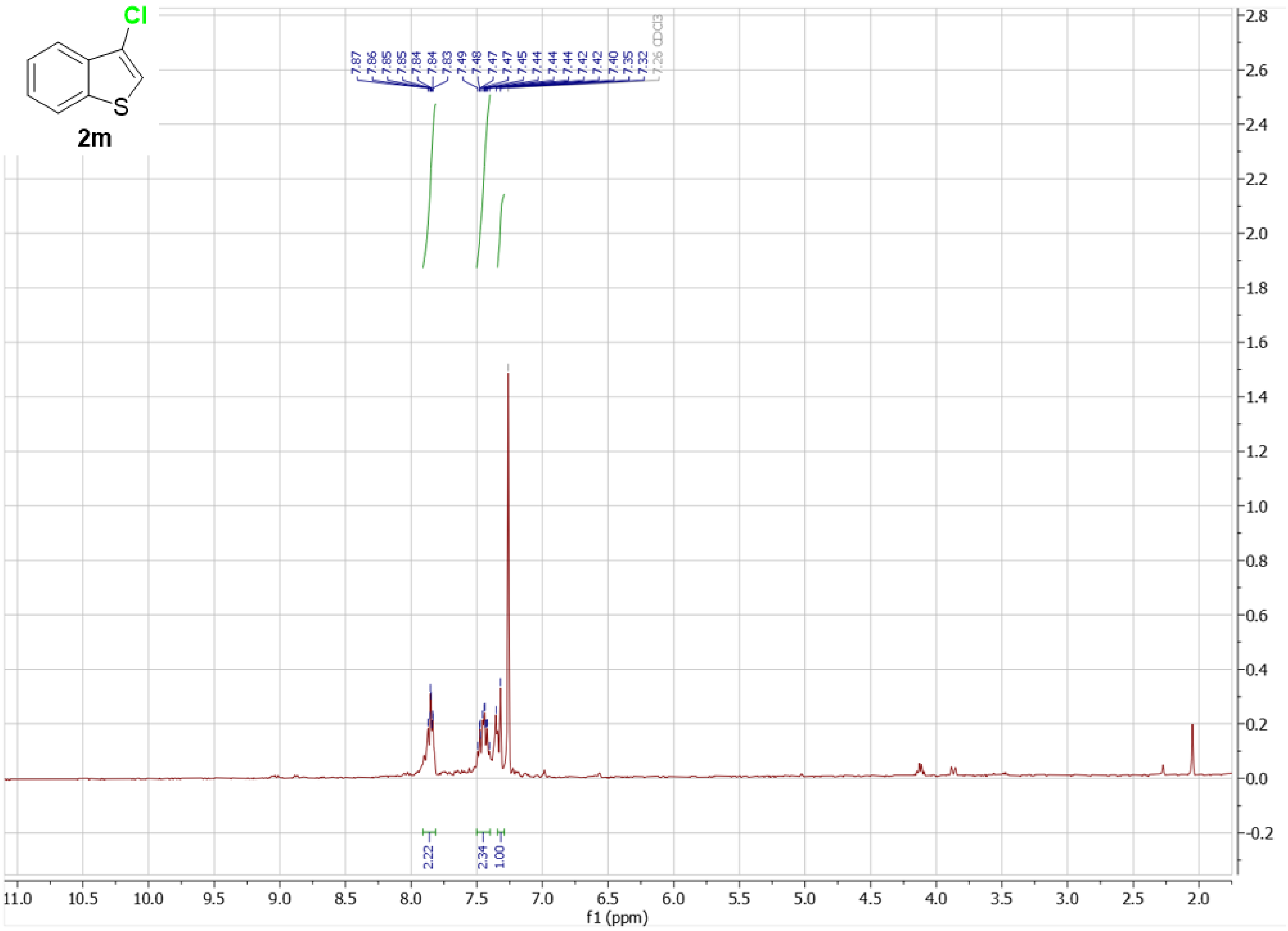

Figure S11. ^{1}H NMR (400 MHz, $CDCl_3$) of **2m**

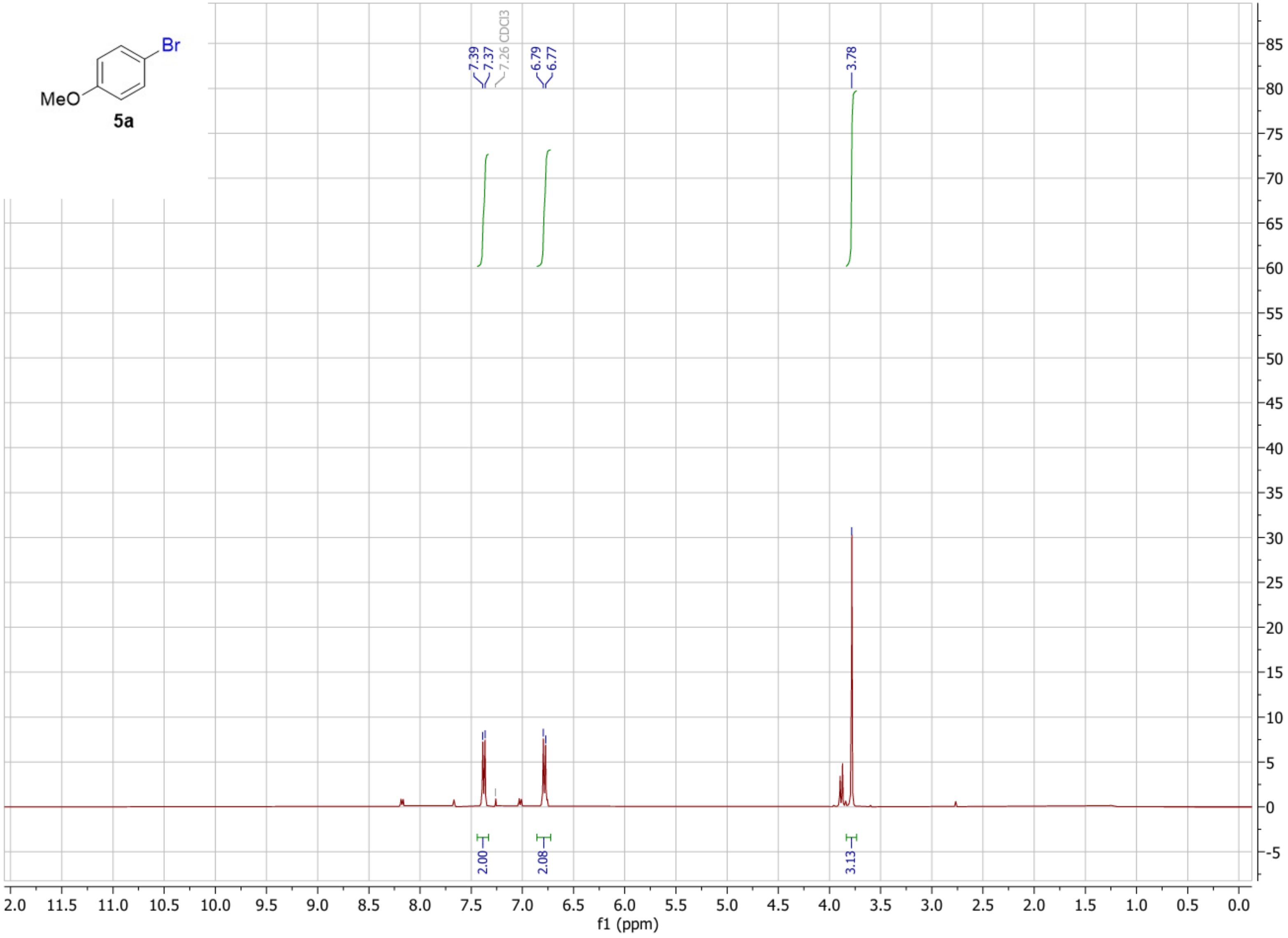

Figure S12. ^{1}H NMR (400 MHz, $CDCl_3$) of **5a**

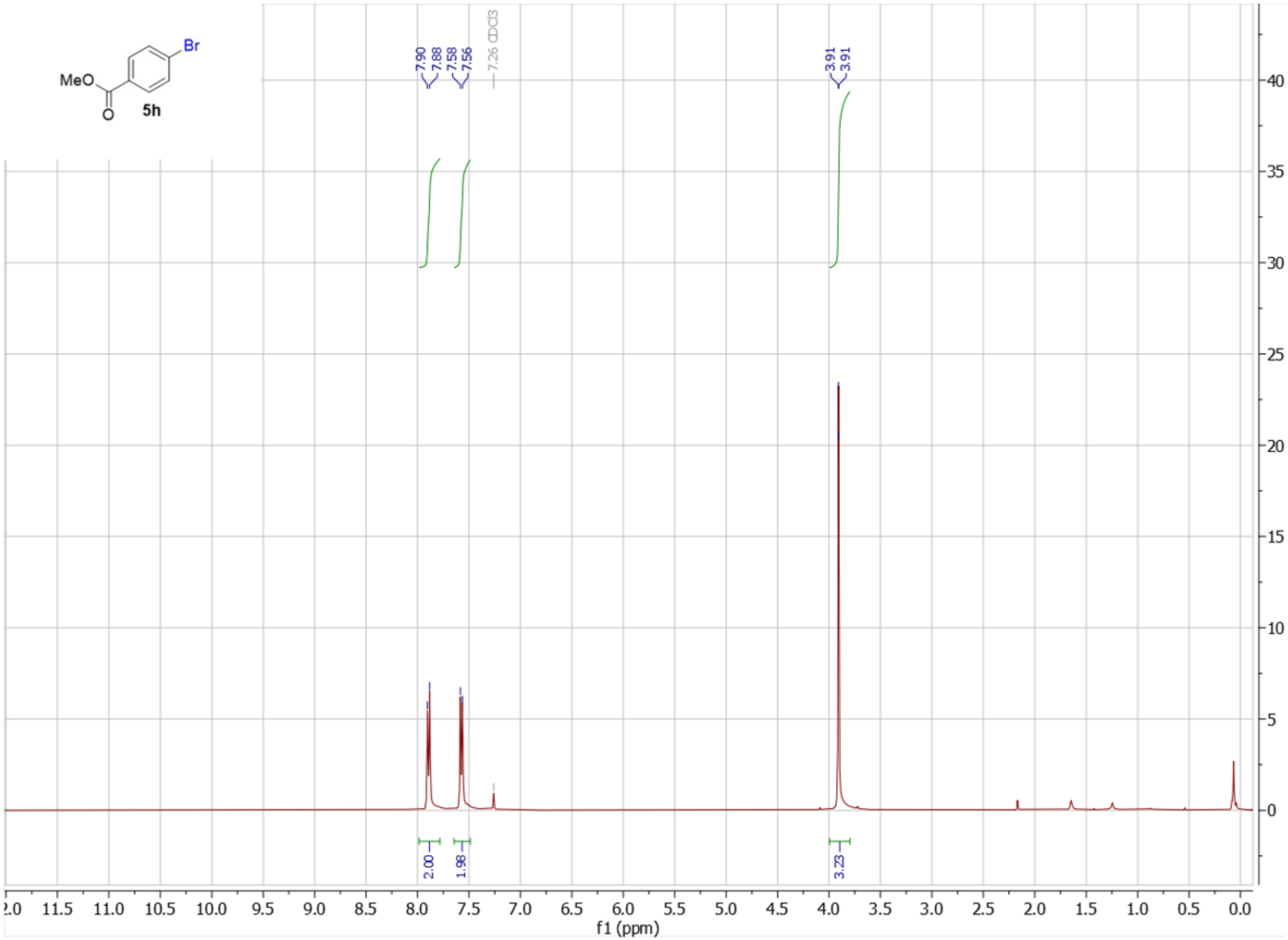

Figure S13. ^{1}H NMR (400 MHz, $CDCl_3$) of **5h**

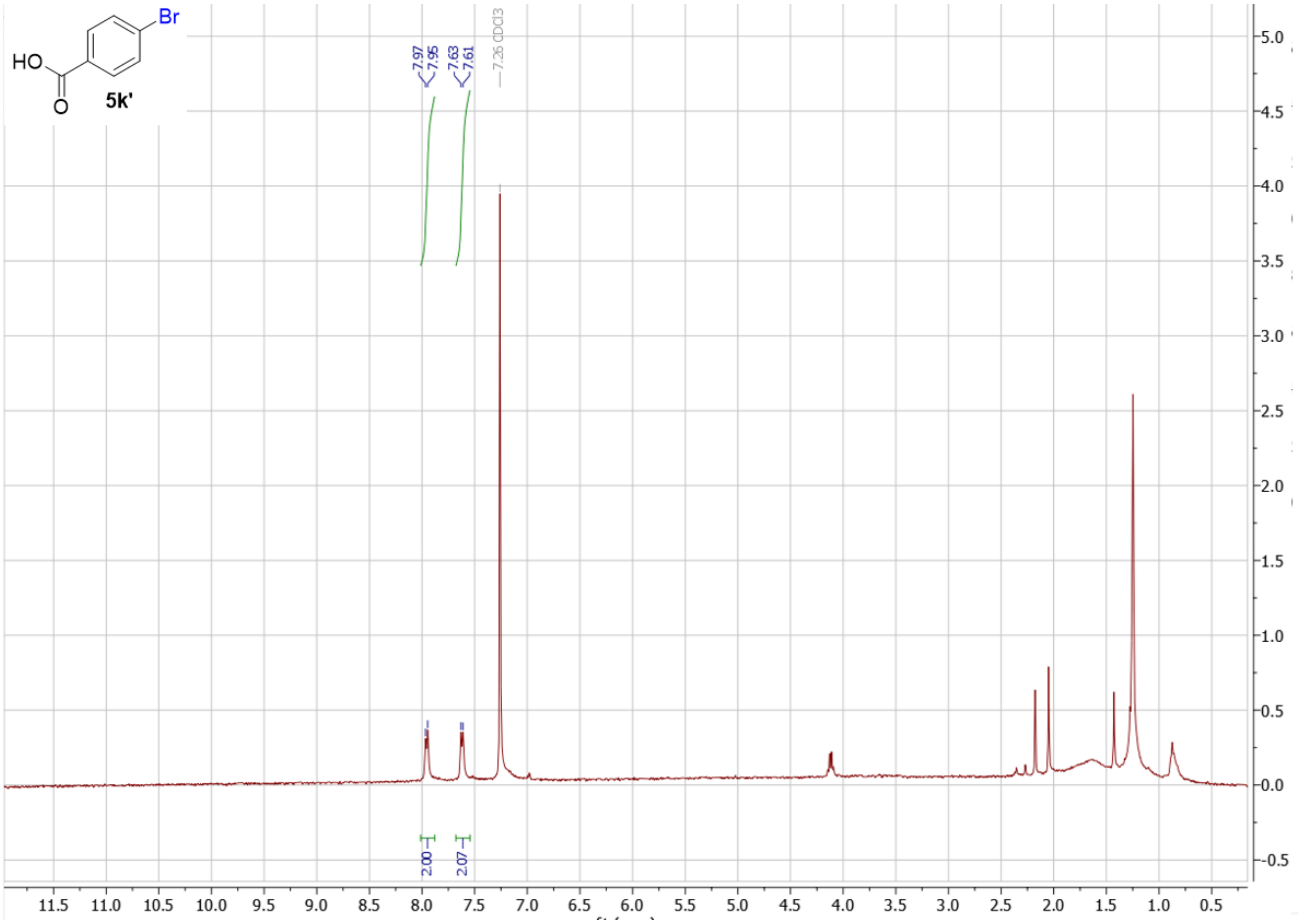

Figure S14. ^{1}H NMR (400 MHz, $CDCl_3$) of **5k’**

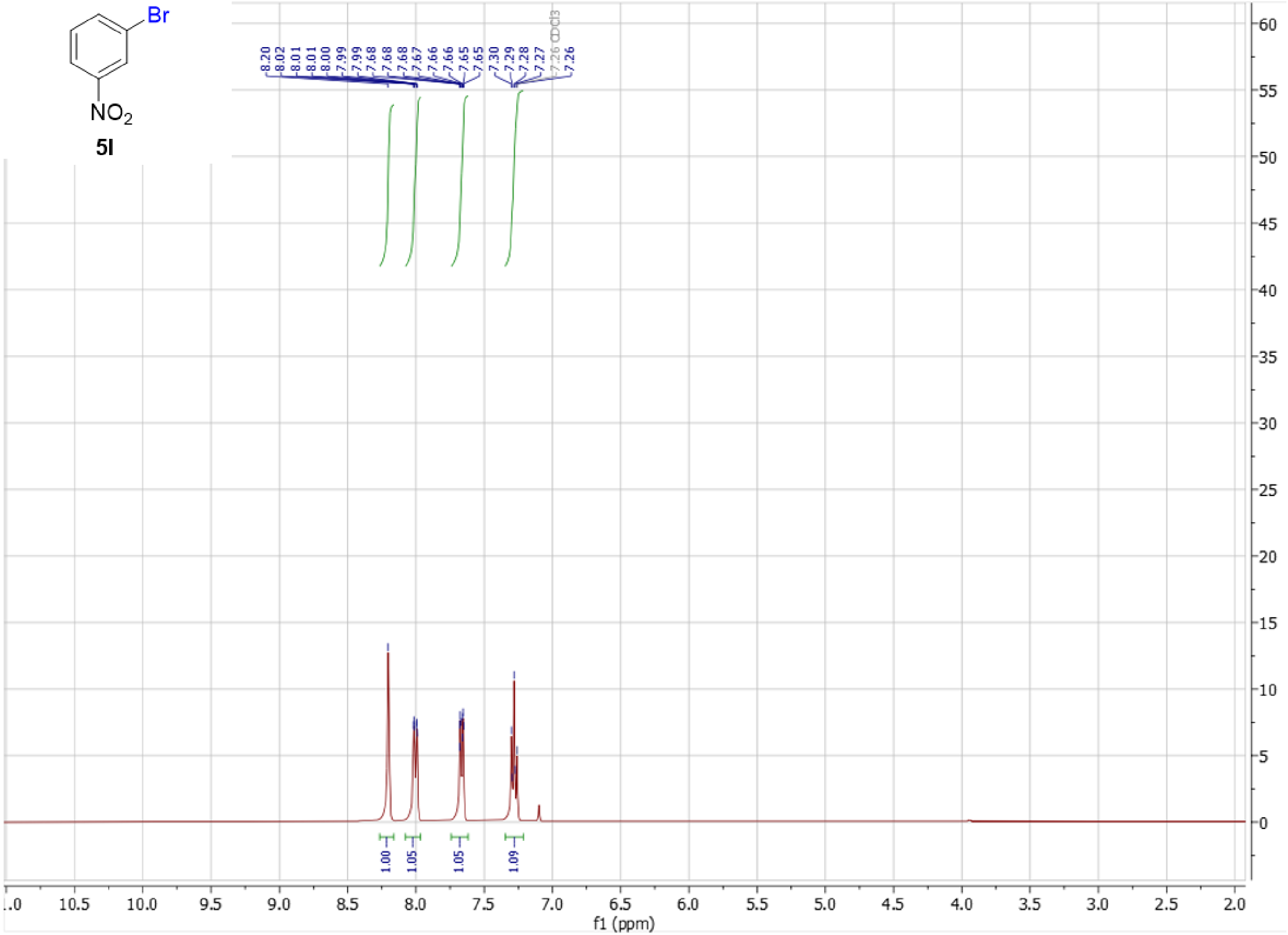

Figure S15. ^{1}H NMR (400 MHz, $CDCl_3$) of **5l**

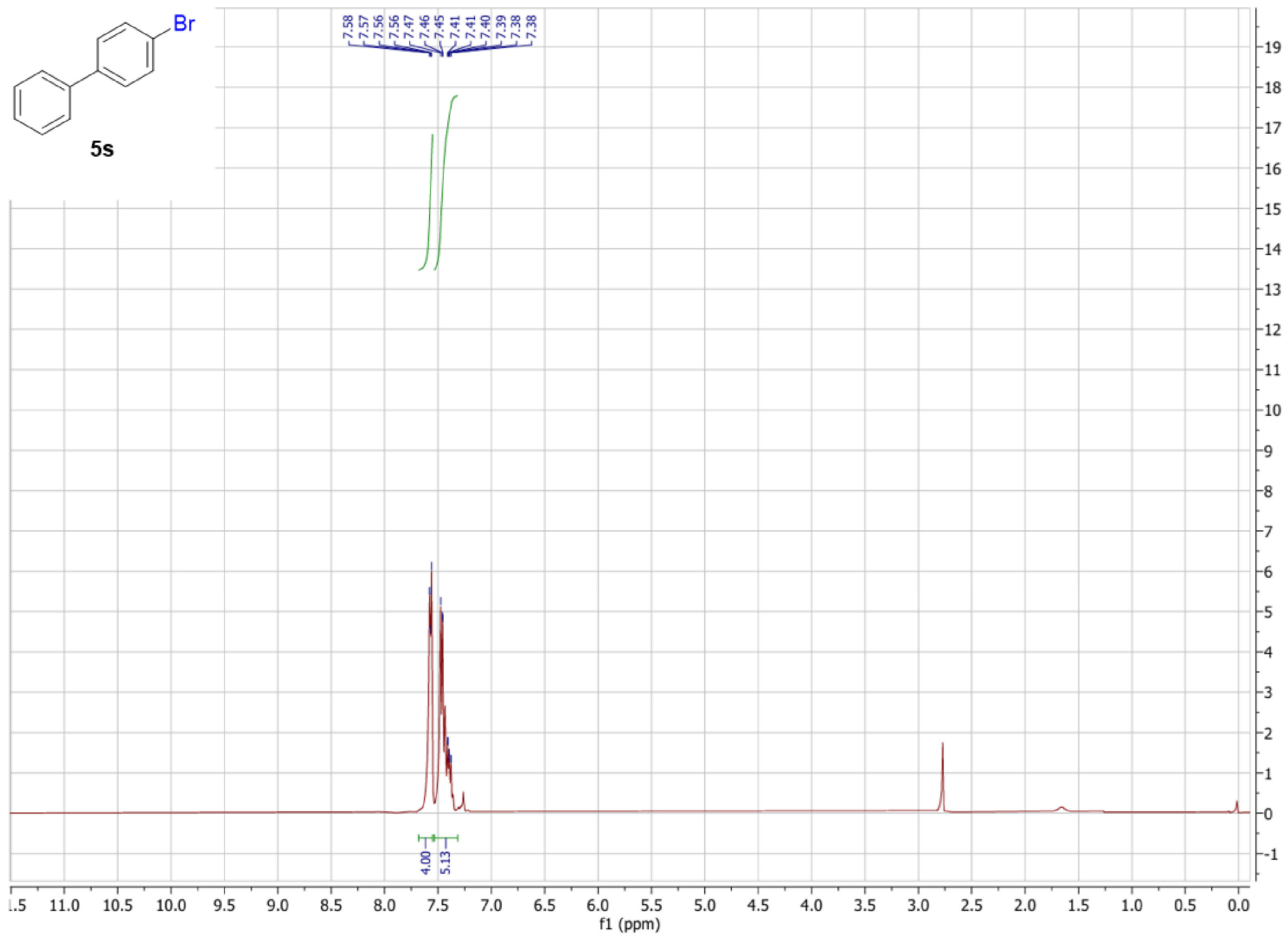

Figure S16. ^{1}H NMR (400 MHz, $CDCl_3$) of **5s**

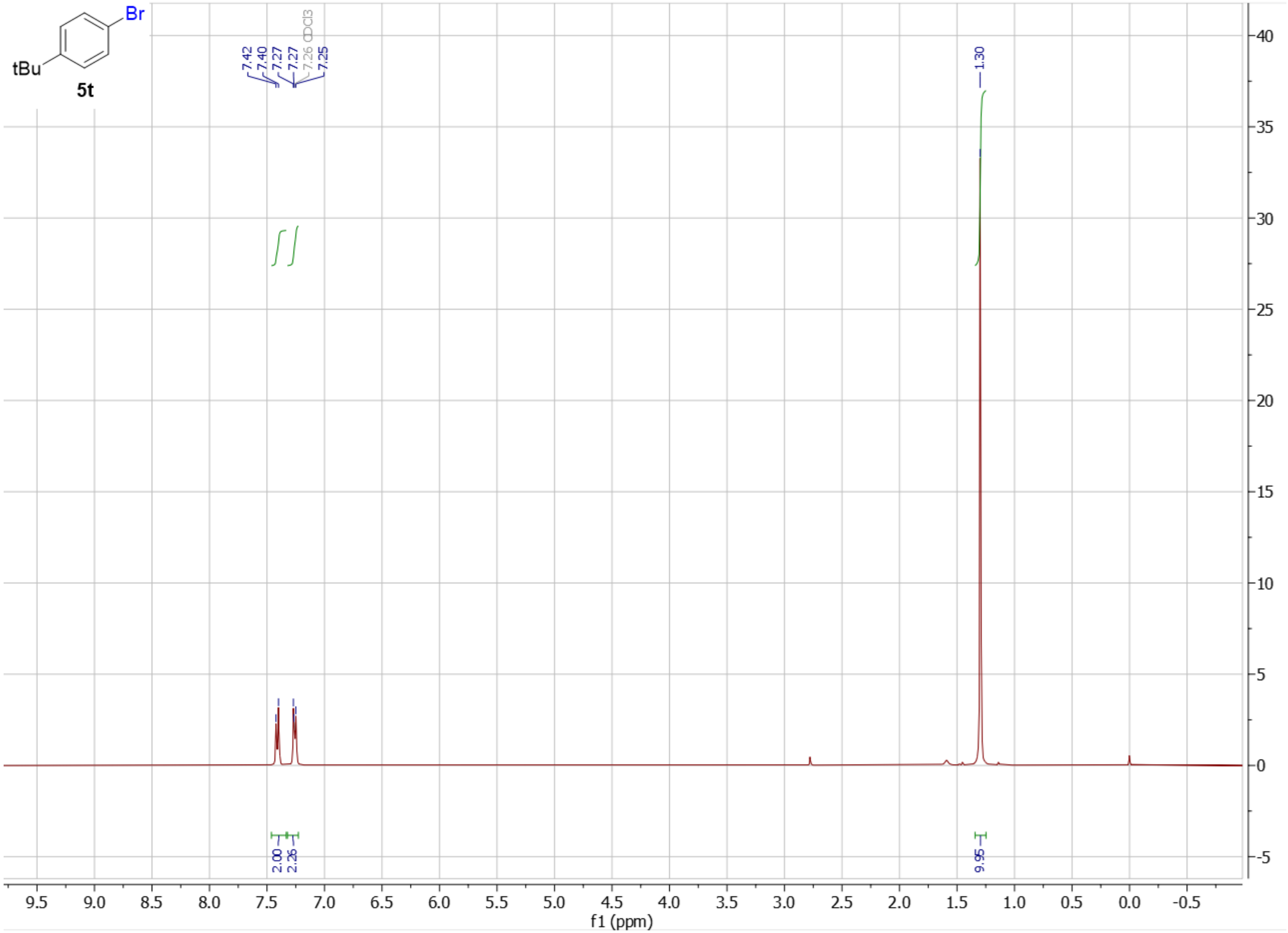

Figure S17. ^{1}H NMR (400 MHz, $CDCl_3$) of **5t**

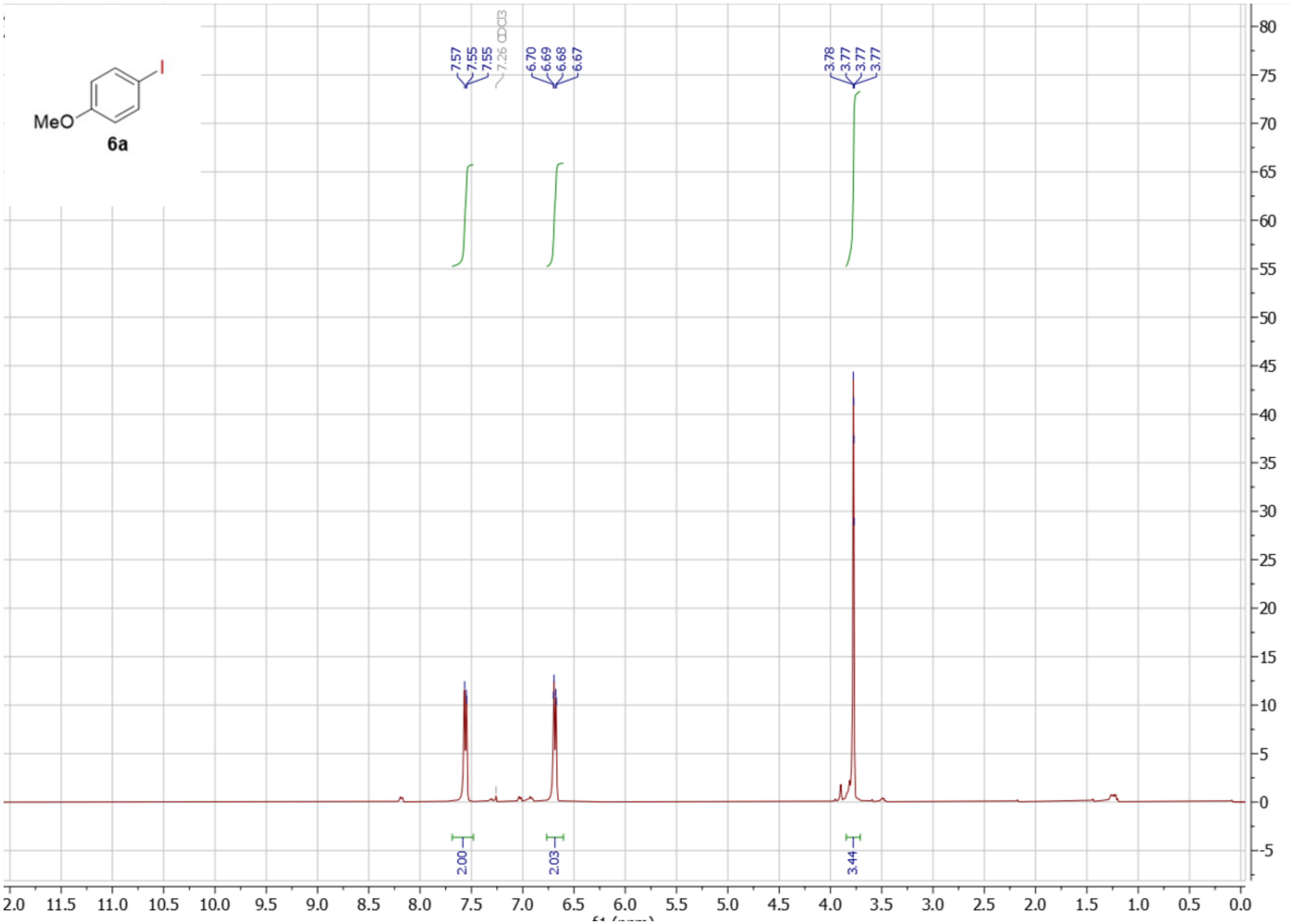

Figure S18. ^{1}H NMR (400 MHz, $CDCl_3$) of **6a**

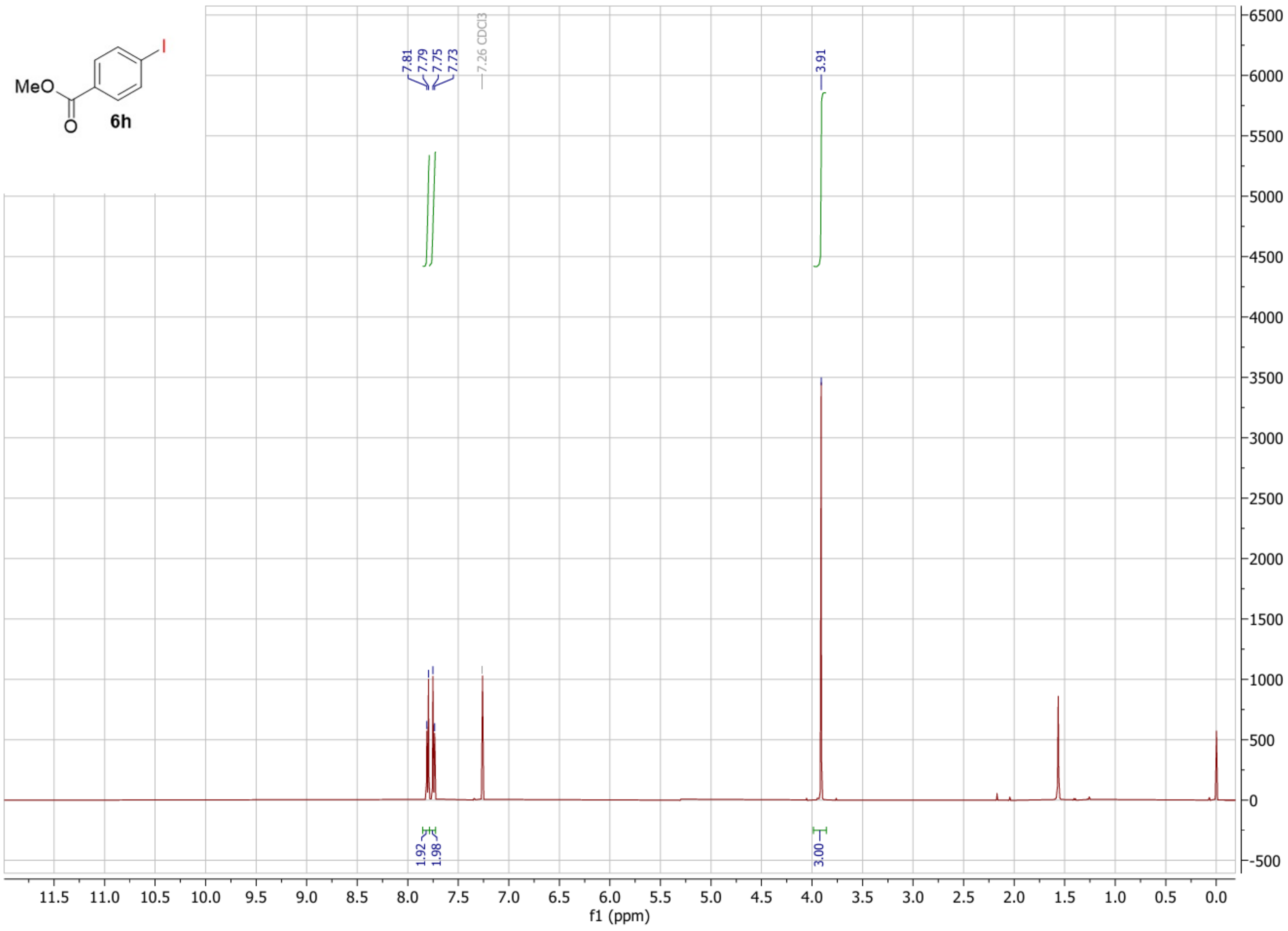

Figure S19. ^{1}H NMR (400 MHz, $CDCl_3$) of **6h**

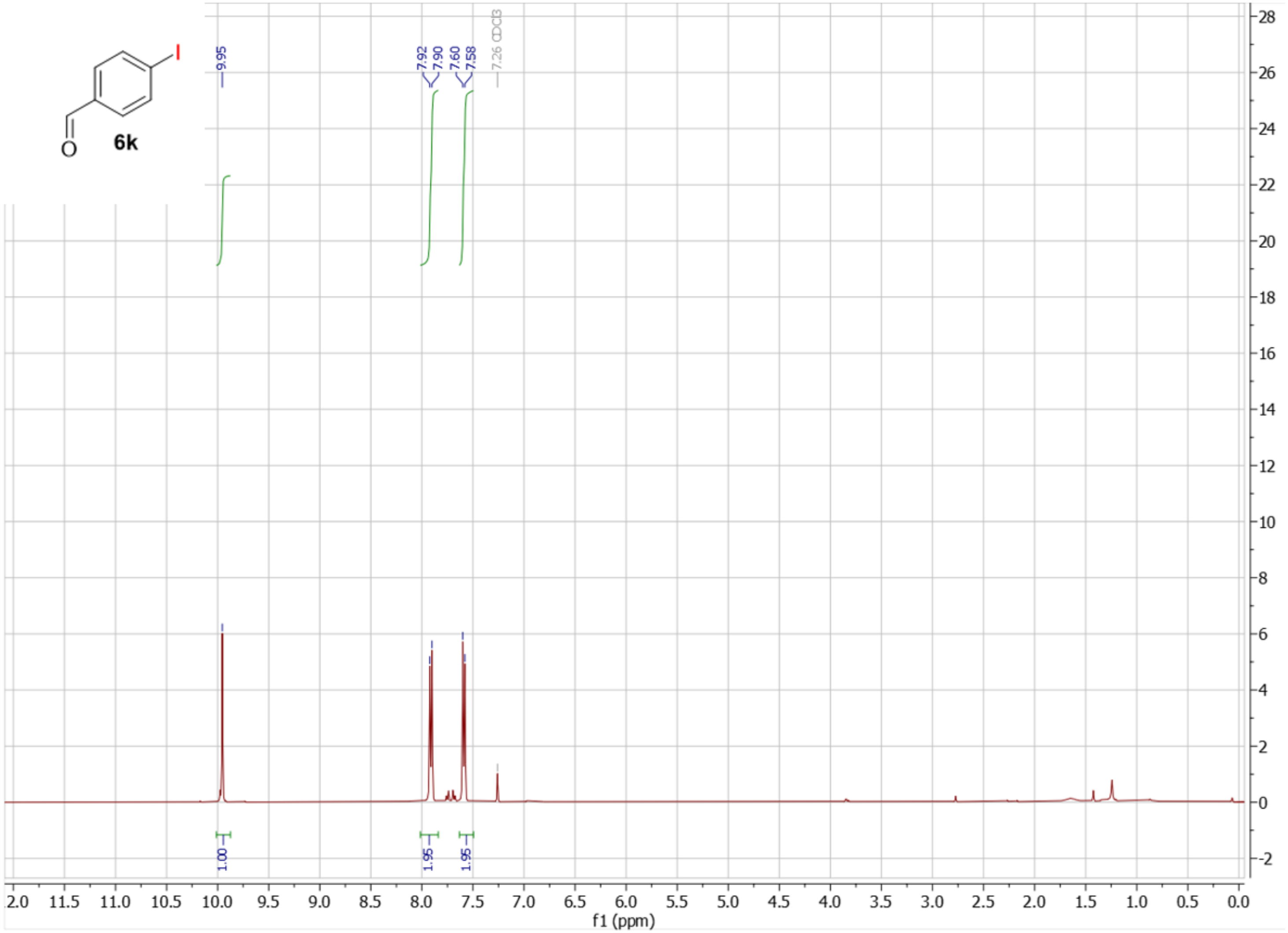

Figure S20. ^{1}H NMR (400 MHz, $CDCl_3$) of **6k**

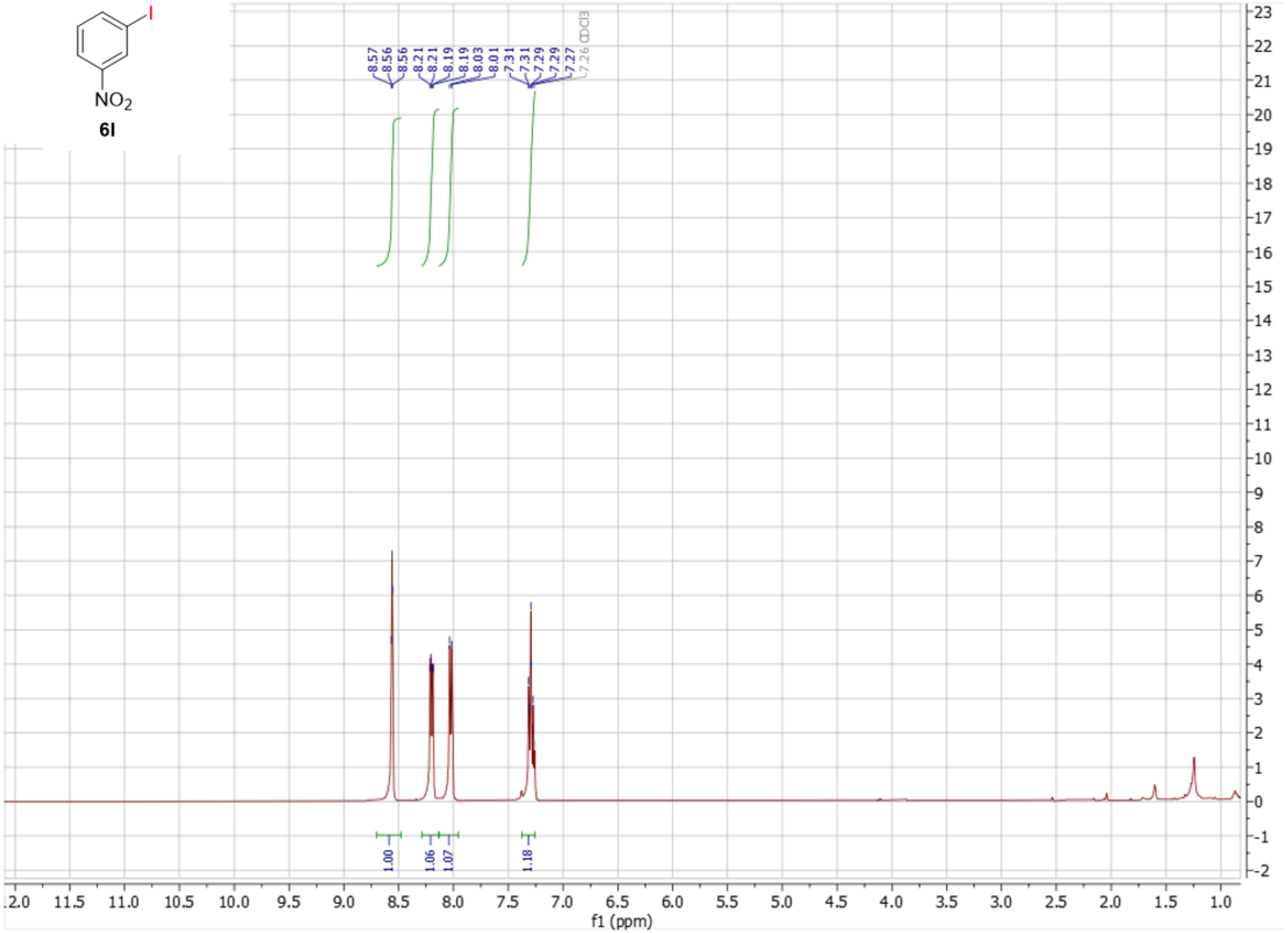

6l

Figure S21. ^{1}H NMR (400 MHz, $CDCl_3$) of **6l**

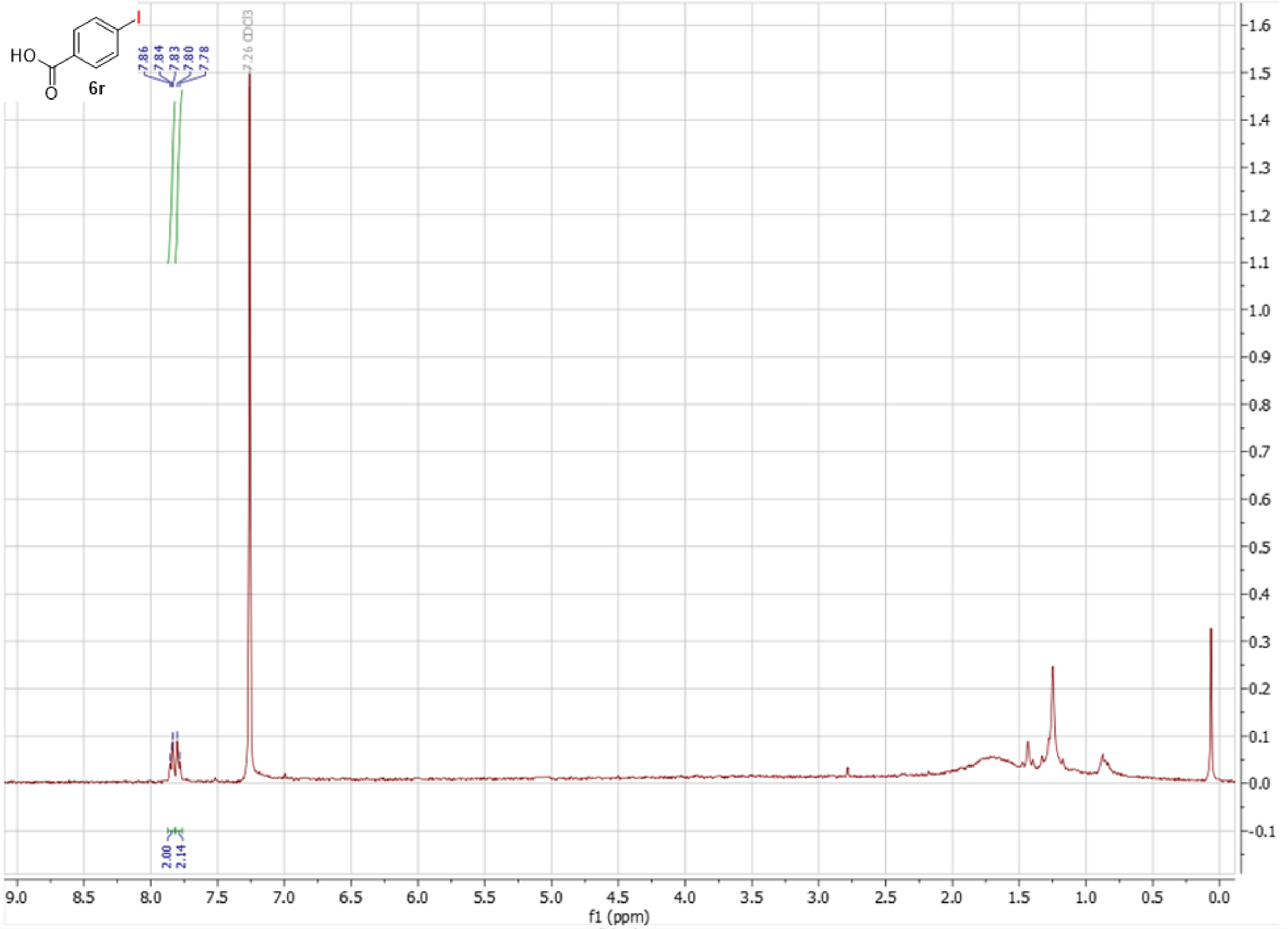

Figure S22. ^{1}H NMR (400 MHz, $CDCl_3$) of **6r**

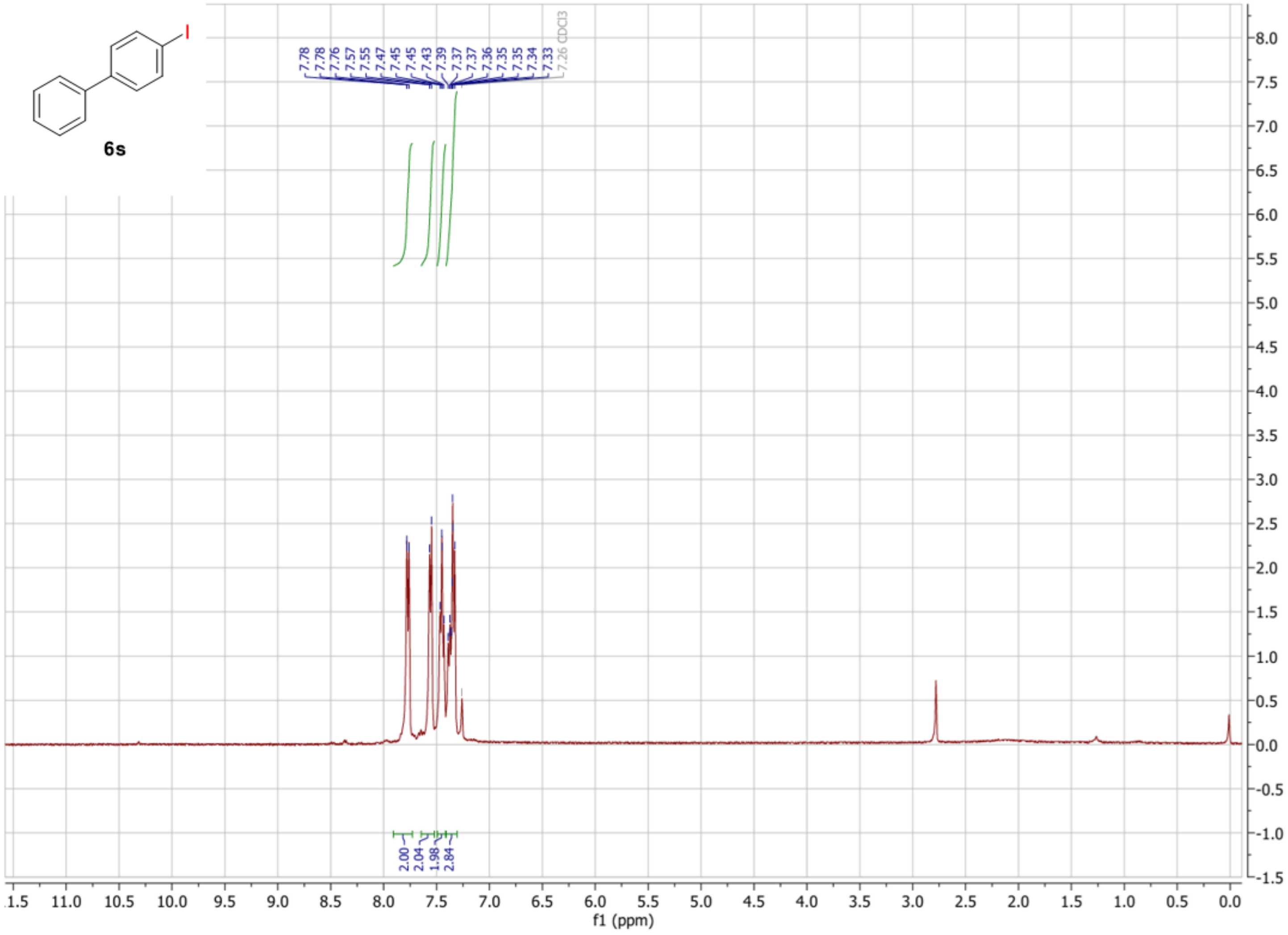

Figure S23. ^{1}H NMR (400 MHz, $CDCl_3$) of **6s**

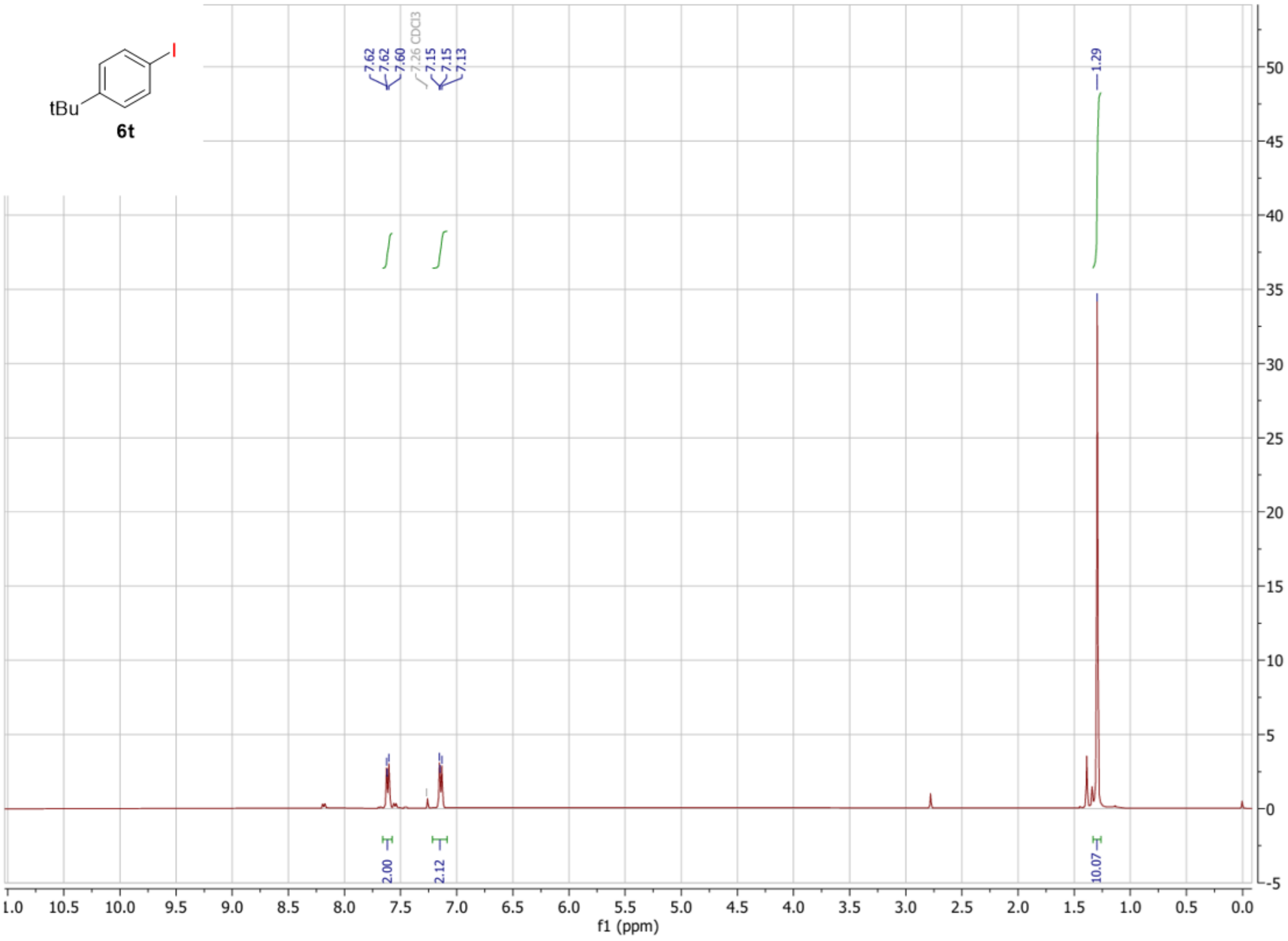

Figure S24. ^{1}H NMR (400 MHz, $CDCl_3$) of **6t**

REFERENCES

1. Cahiez, G.; Moyeux, A.; Gager, O.; Poizat, M. Copper-Catalyzed Decarboxylation of Aromatic Carboxylic Acids: En Route to Milder Reaction Conditions. *Adv. Synth. Catal.* **2013**, 355, 790–796.
2. He, W.; Zhang, R.; Cai, M. A Highly Efficient Heterogeneous Copper-Catalyzed Chlorodeboronation of Arylboronic Acids Leading to Chlorinated Arenes. *RSC Adv.* **2017**, 7, 764–770.
3. Shendage, S. S.; Nagarkar, J. M. Dimethylglyoxime as an Efficient Ligand for Copper-Catalyzed Hydroxylation of Aryl Halides. *J. Chem. Sci.* **2018**, 130, 13.
4. Li, X.; Wei, J.; Wang, W.; Zhang, Y.; Ai, L.; Zhu, Y.; Shi, X.; Zhang, X.; Jiao, N. DMSO-Catalysed Late-Stage Chlorination of (Hetero)Arenes. *Nat. Catal.* **2019**, 3, 107–115.
5. Peng, X.; Shao, X.-F.; Liu, Z.-Q. Pd(II)-Catalyzed Bromo- and Chlorodecarboxylation of Electron-Rich Arenecarboxylic Acids. *Tetrahedron Lett.* **2013**, 54, 3079–3081.
6. Nahide, P. D.; Ramadoss, V.; Juarez Ornelas, K. A.; Satkar, Y.; Ortiz Alvarado, R.; Cervera Villanueva, J. M. J.; Alonso-Castro, Á. J.; Zapata-Morales, J. R.; Ramírez Morales, M. A.; Ruiz-Padilla, A. J.; Deveze Álvarez, M. A.; Solorio-Alvarado, C. R. In Situ Formed I(III)-Based Reagent for the Electrophilic ortho-Chlorination of Phenols and Phenol Ethers: The Use of PIFA-$AlCl_3$ System. *Eur. J. Org. Chem.* **2018**, 2018, 485–493.
7. Leowanawat, P.; Zhang, N.; Safi, M.; Hoffman, D. J.; Fryberger, M. C.; George, A.; Percec, V. *trans*-Chloro(1-Naphthyl)bis(triphenylphosphine)nickel(II)/PCy_3-Catalyzed Cross-Coupling of Aryl and Heteroaryl Neopentylglycolboronates with Aryl and Heteroaryl Mesylates and Sulfamates at Room Temperature. *J. Org. Chem.* **2012**, 77, 2885–2892.
8. Zhang, X.; Zhang, W.-Z.; Shi, L.-L.; Guo, C.-X.; Zhang, L.-L.; Lu, X.-B. Silver(I)-Catalyzed Carboxylation of Arylboronic Esters with CO_2. *Chem. Commun.* **2012**, 48, 6292–6294.
9. Mukhopadhyay, S.; Batra, S. Direct Transformation of Arylamines to Aryl Halides via Sodium Nitrite and N-Halosuccinimide. *Chem. Eur. J.* **2018**, 24, 14622–14626.
10. Guo, T.; Gao, Y.; Li, Z.; Liu, J.; Guo, K. Cyclopropenium-Activated DMSO for Swern-Type Oxidation. *Synlett* **2019**, 30, 329–332.
11. Kuriyama, M.; Hamaguchi, N.; Yano, G.; Tsukuda, K.; Sato, K.; Onomura, O. Deuterodechlorination of Aryl/Heteroaryl Chlorides Catalyzed by a Palladium/Unsymmetrical NHC System. *J. Org. Chem.* **2016**, 81, 8934–8946.
12. Bovonsombat, P.; Teecomegaet, P.; Kulvaranon, P.; Pandey, A.; Chobtumskul, K.; Tungsirisurp, S.; Sophanpanichkul, P.; Losuwanakul, S.; Soimaneewan, D.; Kanjanwongpaisan, P.; Siricharoensang, P.; Choosakoonkriang, S. Regioselective Monobromination of Aromatics via a Halogen Bond Acceptor-Donor Interaction of Catalytic Thioamide and N-Bromosuccinimide. *Tetrahedron* **2017**, 73, 6564–6572.
13. Fu, Z.; Hao, G.; Fu, Y.; He, D.; Tuo, X.; Guo, S.; Cai, H. Transition Metal-Free Electrocatalytic Halodeborylation of Arylboronic Acids with Metal Halides MX (X = I, Br) to Synthesize Aryl Halides. *Org. Chem. Front.* **2020**, 7, 590–595.

14. Tran, D. P.; Nomoto, A.; Mita, S.; Dong, C.-P.; Kodama, S.; Mizuno, T.; Ogawa, A. Metal- and Base-Free Synthesis of Aryl Bromides from Arylhydrazines. Tetrahedron Lett. **2020**, 61, 151959.
15. Wan, J.-P.; Wang, C.; Zhou, R.; Liu, Y. Sustainable H_2O/Ethyl Lactate System for Ligand-Free Suzuki–Miyaura Reaction. *RSC Adv.* **2012**, 2, 8789-8792
16. Yao, M.-L.; Reddy, M. S.; Yong, L.; Walfish, I.; Blevins, D. W.; Kabalka, G. W. Chemoselective Bromodeboronation of Organotrifluoroborates Using Tetrabutylammonium Tribromide: Application in (Z)-Dibromoalkene Syntheses. *Org. Lett.* **2010**, 12, 700–703.
17. Zhang, J.; Li, S.; Deng, G.; Gong, H. Metal-Free, Oxidant-Free, and Controllable Graphene Oxide Catalyzed Direct Iodination of Arenes and Ketones. *ChemCatChem* **2017**, 10, 376–380.
18. Babu, S. S.; Shanmugam, S. CAN-Supported Chemoselective Oxidative Conversion of α-Aroylketene-(S,S)-Acetals to Aryl Carboxylic Acids. *ChemistrySelect* **2017**, 2, 2330–2334.
19. Tinnis, F.; Volkov, A.; Slagbrand, T.; Adolfsson, H. Chemoselective Reduction of Tertiary Amides under Thermal Control: Formation of Either Aldehydes or Amines. *Angew. Chem. Int. Ed*. **2016**, 55, 4562–4566.
20. Yang, H.; Li, Y.; Jiang, M.; Wang, J.; Fu, H. General Copper-Catalyzed Transformations of Functional Groups from Arylboronic Acids in Water. *Chem. Eur. J.* **2011**, 17, 5652–5660.
21. Liu, W.; Liu, P.; Lv, L.; Li, C.-J. Metal-Free and Redox-Neutral Conversion of Organotrifluoroborates to Radicals Enabled by Visible Light. *Angew. Chem. Int. Ed.* **2018**, 57, 11458–11462.
22. Liu, S.; Chen, J.; Zhang, R.; Zhao, F.; Deng, G.-J. Palladium-Catalyzed Desulfinative Iodination of Sodium Sulfinates and Sulfonyl Hydrazides. *Asian J. Org. Chem.* **2014**, 3, 1150–1153.
